# A Smart Semi‐Implantable Device Integrating Microchannel‐Enhanced Sampling and Multiplex Biochemical Testing for Deep Wound Monitoring and Pathogen Identification

**DOI:** 10.1002/advs.202407868

**Published:** 2024-12-31

**Authors:** Qilin Li, Chunyu Wei, Luming Xu, Jiao Zhang, Yuyu Li, Xiaohuan Lu, Rengui Xu, Honglian Guo, Peng Cao, Chenke Ouyang, Jiarong Xu, Wei Chen, Zheng Wang, Lin Wang

**Affiliations:** ^1^ Department of Clinical Laboratory Union Hospital Tongji Medical College Huazhong University of Science and Technology Wuhan 430022 China; ^2^ Hubei Key Laboratory of Regenerative Medicine and Multi‐disciplinary Translational Research Hubei Provincial Engineering Research Center of Clinical Laboratory and Active Health Smart Equipment Research Center for Tissue Engineering and Regenerative Medicine Union Hospital Tongji Medical College Huazhong University of Science and Technology Wuhan 430022 China; ^3^ Department of Gastrointestinal Surgery Union Hospital Tongji Medical College Huazhong University of Science and Technology Wuhan 430022 China; ^4^ Department of Pharmacology School of Basic Medicine State Key Laboratory for Diagnosis and Treatment of Severe Zoonotic Infectious Diseases Tongji‐Rongcheng Center for Biomedicine Tongji Medical College Huazhong University of Science and Technology Hubei Key Laboratory for Drug Target Research and Pharmacodynamic Evaluation Huazhong University of Science and Technology Wuhan 430030 China

**Keywords:** deep sampling, deep wound monitoring, multiplex biochemical testing, pathogen identification, smart semi‐implantable device

## Abstract

Monitoring deep wounds is challenging but necessary for high‐quality medical treatment. Current methodologies for deep wound monitoring are typically limited to indirect clinical symptoms or costly non‐real‐time imaging diagnosis. Herein, a smart system is proposed that enables in situ monitoring of deep wounds’ status through a semi‐implantable device composed of 2 seamlessly connected functional components: 1) the well‐designed, microchannel‐structured sampling needles that efficiently and conveniently collect samples from deep wound anatomical locations, and 2) the multiplex biochemical testing compartment that facilitates the immediate and persistent detection of multiple biochemical indicators based on a color image processing software accessible to a conventional smartphone. With the 3 representative preclinical deep wound models, the study demonstrates the device's potential to monitor wound infection, inflammation, healing progress, and reduce inflammation when applied to deep skin injury, surgical implantation, and postoperative intestinal leakage. The device's capability to rapidly and accurately identify pathogenic bacteria is also demonstrated both in vitro and in vivo, potentially facilitating precise intervention in infected wounds. Coupled with the device's favorable biocompatibility and cost‐effectiveness, this intelligent system emerges as a promising tool for safe and effective management of complicated deep wounds.

## Graphical abstract

Monitoring deep wounds is critical but challenging. This study introduces a semi‐implantable smart system integrating surface microchannel‐structured sampling needles and a multiplex biochemical testing compartment. It enables continuous detection of infection, inflammation, and healing progress in deep wounds via smartphone‐compatible colorimetric analysis. Demonstrating biocompatibility, cost‐effectiveness, and precision in preclinical models, this system offers a promising approach to managing complex wounds.

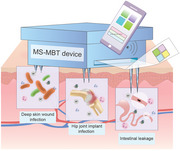

## 1 Introduction

Deep wounds, prevalently encountered in surgical procedures, traumatic injury, and chronic diseases, require accurate and continuous monitoring, given the high risk of developing severe complications that could greatly hinder the healing process and even increase mortality. For instance, deep surgical wounds, such as surgical incisions into deep tissues (such as gastrointestinal and genitourinary tracts) and internal organ transplantation, are prone to a range of complications, including infection, bleeding, obstruction, and leakage, thereby increasing the complexity of medical management.^[^ ^1^ ^]^ These complications also occur even in minimally invasive surgery but are preventable or manageable with timely recognition and proper postoperative care.^[^ ^2^ ^]^ Clinically, postoperative complications related to deep surgical wounds are primarily diagnosed by patients’ symptoms (e.g., pain, nausea, and vomiting), vital signs (e.g., temperature, respiration, heart rate, and blood pressure), and/or laboratory examination on peripheral blood (blood routine, biochemical, and immunological tests). However, these indirect indicators often do not offer a timely representation of actual deep wound status; when they are abnormal, complications have often escalated to a serious level, thereby missing the proper timing of medical intervention.^[^ ^3^ ^]^ Imaging and endoscopic diagnostics are of clinical utility for visualizing deep wounds,^[^ ^1^, ^3^, ^4^ ^]^ but require expensive equipment and professionally trained medical personnel for instrumental operation and data interpretation, often prolonging the time period to produce results and not readily accessible for patients’ post‐discharge monitoring. Additionally, frequent radiation exposure and invasive endoscopy may generate serious adverse effects, such as skin allergy, blood system abnormalities, tumors, and organ injury, thus unsuitable for long‐term monitoring.^[^ ^5^ ^]^

Physical and chemical analysis of samples directly from wound sites may offer a more accurate and in‐time assessment of wound status since the physical characteristics of wound tissues and the biochemical composition of wound exudate provide valuable information regarding wound types, microbial infection, and healing stages.^[^ ^6^ ^]^ Although the conventional approaches (e.g., swabbing) and the newly‐emerged wearable devices that finely embed patches, dressings, or bandages with electrical and electrochemical sensors are promising to transform wound management to be more timely and accessible to point‐of‐care and personalized service,^[^ ^6^ ^]^ most of these approaches and devices are merely effective for superficial wounds (including abrasions, lacerations, burn, and chronic condition‐related injuries affecting the epidermis and, in certain instances, the dermis), but not suitable for monitoring deep and/or closed wounds.^[^ ^7^ ^]^ This is largely due to a lack of effective sampling and monitoring techniques for deep wounds, which also hinders the revelation of biomarkers that can precisely reflect diverse statuses of deep wounds.

To access the wound sites, clinical fine‐needle aspiration and core needle biopsy facilitate the penetration into the skin to access deep anatomic sites for liquid or tissue sample collection.^[^ ^8^ ^]^ However, these invasive procedures generally necessitate skilled medical personnel. Moreover, clinical sampling and testing procedures often involve a series of disconnected steps, from patient sample collection, transportation, processing, and laboratory testing, to subsequent analysis. This fragmented workflow can not only delay medical intervention but also heighten the likelihood of sample contamination and erroneous results. Alternatively, integrating sampling and testing steps can substantially improve the efficiency of clinical management by highly streamlining the workflow, saving time and costs, as well as reducing the risk of sample contamination, thus being particularly suitable for point‐of‐care monitoring in resource‐limited and post‐discharge settings.^[^ ^9^ ^]^

Compared to stainless‐steel needles, the recent utilization of needles made of materials with lower stiffness, such as polymers, may minimize tissue damage and mitigate patient discomfort during and post usage.^[^ ^10^ ^]^ With the help of 3D printing technology, microneedles generally arrayed on a base layer can be fabricated using various polymer materials (e.g., resin, gelatin, and alginate), and offer a minimally invasive, painless, and self‐administrated alternative to conventional hypodermic needles.^[^ ^10b^ ^]^ Microneedles can collect interstitial fluid (ISF) or blood for the in vitro detection of biomolecules, and also facilitate in situ and swift detection of local biomarkers when integrated with specific detection systems.^[^ ^11^ ^]^ However, conventional microneedles, typically less than 1 mm in length, are constrained in sample collection from shallow skin layers. Moreover, limited sampling efficiency remains a crucial obstacle in the development of microneedles for in vivo medical detection.^[^ ^12^ ^]^ Although an external vacuum device was proposed to enhance fluid extraction^[^ ^11^, ^13^ ^]^ it further complicated the sampling process and hindered the integration of testing units. Hollow and porous microneedles reportedly extract more fluid samples than solid microneedles, but they often tend to be compressed by tissues, leading to irreversible sample clogging.^[^ ^14^ ^]^

**Scheme 1:**
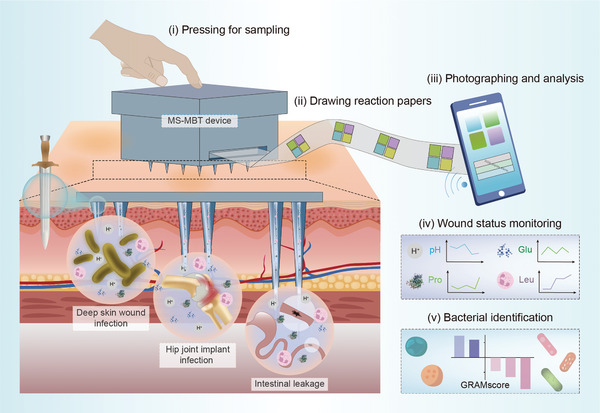
Schematic illustration of the MS‐MBT device that enables monitoring of diverse deep wounds and identification of multiple pathogens through microchannel‐enhanced sampling and multiplex biochemical testing. i) Pressing onto the device for collecting the fluid samples from the deep wound locations through the longsword fuller‐inspired microchannels on the sampling needles’ surface. ii) Continuously drawing the reaction papers for immediate and persistent biochemical testing. iii) Quantitative analysis of the biochemical indicators using a smartphone integrated with ColorPicker software. iv) Monitoring of the wound status by in situ collection and detection of the multiple biochemical indicators with the device. v) Indentation of various pathogenic bacteria using the device coupled with machine learning models.

Herein, we designed and fabricated a miniaturized semi‐implantable device integrated with both sampling and biochemical testing units for early detection and continuous monitoring of deep infected wounds to facilitate clinical management and home care of diverse wounds (**Scheme** **1**). The sampling unit consists of tiny resin needles featured with microchannels on the surface inspired by the medieval longsword with the central fuller running along the blade and arrayed with oval tunnels for sample collection. We demonstrated that the needles were sufficiently rigid to penetrate the skin and reached deep locations (ranging from 2 to 10 mm) without inducing severe tissue inflammation or leaving permanent wounds upon removal. Unlike conventional hollow sampling needles that rely on strong negative pressure mechanisms to aspirate samples, this device allows for efficient collection of biological fluids from deep tissues or body cavities through enhanced capillarity by simply applying finger pressure onto the device. This feature facilitated effective functional cooperation with the testing unit, where fluid was aspirated up into the testing compartment along needles’ microchannels and oval tunnels for specific chemical reactions. The testing compartment shielded with a spring cap housed continuously withdrawable chemical reaction paper strips for immediate and persistent multiplex biochemical examination. Moreover, the quantification of the biochemicals of interest could be conveniently performed using a smartphone installed with a self‐developed image processing software (ColorPicker) based on automatic colorimetric feature extraction, color value calculation, and variance analysis‐based data mapping. This highly integrated smart system, with tunable physical parameters of sampling needles and customizable biochemical indicator panels, provides ample opportunities to meet broad needs for monitoring diverse wound types. Given that wound infection, inflammation, and healing process involve the participation of diverse biological factors,^[^ ^6^, ^15^ ^]^ we opted for a multilevel wound biomarker set encompassing pH, glucose (Glu), protein (Pro), and leukocytes (Leu) to acquire a comprehensive profile of different wounds’ status.

The feasibility of the microchannel‐enhanced sampling and multiplex biochemical testing (MS‐MBT) device for rapid diagnosis and continuous monitoring of deep complicated wounds was demonstrated using tissue‐mimicking gel, isolated porcine skin tissue, and 3 preclinical wound models in rats, including bacteria‐infected deep skin wounds, hip joint implants, and postoperative intestinal leakage. Our investigations revealed that this device greatly facilitated the early detection of wound infection/intestinal leakage, assessment of their severity, tracking of wound inflammation and healing progress, as well as providing helpful guidance for anti‐inflammatory treatments. Furthermore, to enhance the device's potential to promote precise and personalized management of infected wounds, we screened 3 biochemical indicators associated with bacterial infection (pH, horse radish peroxidase (HRP), and specific gravity (SG)), and demonstrated that the MS‐MBT device collecting and detecting these indicators allowed for the identification of pathogenic bacteria commonly accounting for accidental and surgical wound infections both in vitro and in vivo with the assistance of machine learning. Thus, this device would be capable of offering timely guidance for targeted medication and possibly preventing antibiotic abuse.

## 2 Results

### 2.1 Preparation and Characterization of MS‐MBT Device

**Figure 1:**
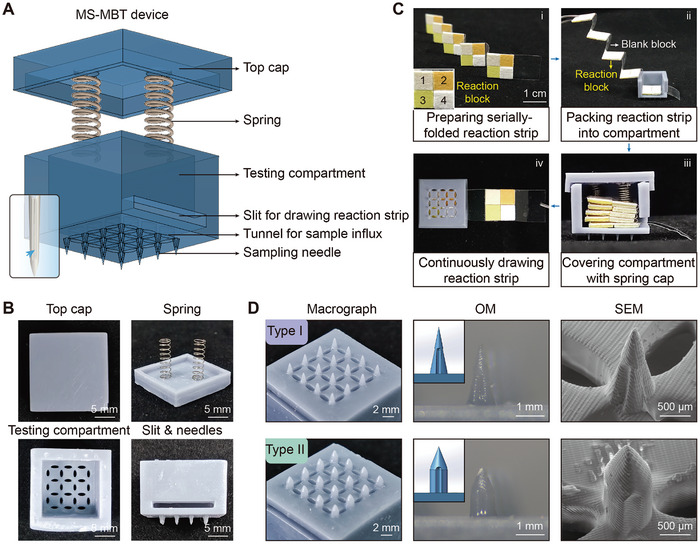
Preparation and morphological characterization of the MS‐MBT device. A) Schematics presenting the device's components including the spring cap and testing compartment on which sword groove (blue arrow)‐inspired needles and oval sample inlets were arrayed. B) Images showing the individual segments of the device. C) The process of the reaction paper encasement and extraction. D) Macrograph, optical microscopy (OM), and scanning electron microscopy (SEM) images of the type I and type II needles. The insets in OM images, the models designed in the Solidworks software.

The MS‐MBT device consisted of 2 main structural components, including the top cap and the testing compartment with longsword fuller‐inspired sampling needles (**Figure** **1**A,B). These components were designed in the Solidworks software and separately printed using UV‐curable resin as the raw material that exhibits excellent biocompatibility, thermostability, mechanical strength, and degradability.^[^ ^10b^ ^]^ Two stainless‐steel springs, with a length of 10 mm, a wire diameter of 0.2 mm, and an outer diameter of 3 mm, were specially installed on the cap's inner surface to facilitate the fixation and continuous extraction of reaction papers (Figure 1A,B). Four individual dry‐chemical reaction papers (2 × 2) were affixed onto the waterproof strip (110 × 11 × 70 µm) to constitute a reaction block (11 × 11 mm) (Figure 1C), which was then loaded into the testing compartment with the reaction papers facing down and folded alternately with a blank waterproof strip block to prevent cross‐contamination between sequential reaction paper blocks (Figure 1C; Figure S1 and Video S1, Supporting Information). The reaction paper blocks could be continuously pulled from the testing compartment's slit for persistent biochemical detection (Figure 1C; Video S1, Supporting Information). The base of the testing compartment was systematically distributed with the needles possessing axial surface channels and oval‐shaped tunnels (Figure 1D), which were coordinated to facilitate the flow of fluid samples and their entry into the testing compartment for the infiltration and specific reaction with reaction papers (details presented in the following sections). Benefiting from the integrated yet spatially separated sampling and testing modules, continuous wound monitoring could be achieved. Once the sampling needles are inserted and the device is fixed in position, only the reaction block within the testing compartment requires replacement according to the number of test days needed per monitoring period.

**Figure 2:**
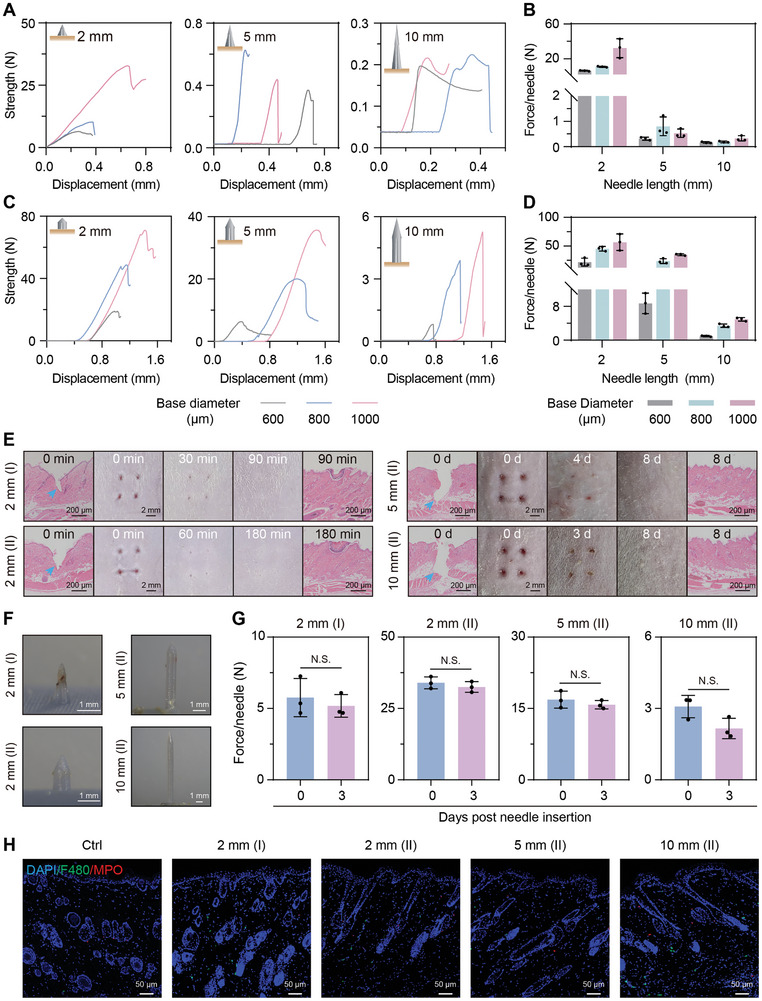
Mechanical performance and biosafety of the MS‐MBT device. A–D) Compressive strength (A, C) and compressive failure force (B, D) of type I (A, B) and type II (C, D) needles with different base diameters and needle lengths. E) The hematoxylin and eosin (H&E) staining and photographic images showing the rat dorsal skin after treatment by different sampling needles. The blue arrows point to the microchannels produced by the needle insertion. F) Images showing the sample needles removed from rats' dorsal tissue 3 days after insertion. G) Compressive failure force of the sample needles before and after insertion into rats' dorsal tissue for 3 days. H) Representative immunofluorescence staining images (merged) of neutrophils (MPO^+^, red) and macrophages (F4/80^+^, green) within the rats’ dorsal tissues 3 days after needle removal. DAPI, nuclei. Data are presented as mean ± SD. N.S., not significant; Student's *t*‐tests.

### 2.2 Highly Tunable Mechanical Performance and Minimally Invasive Tissue Penetration

Given the variability in tissue hardness and location depths of wounds arising from diverse causes, we devised 2 distinct needle shapes, a conical type (type I) and a cone‐cylinder type (type II) (Figure 1D), with tunable lengths and base diameters. Specifically, the type I needle was relatively sharp but fragile, thus puncturing soft tissue with minimal invasion, while the type II needle held greater mechanical strength due to its thicker needle trunk, making it more suitable for tough tissue. Moreover, the needle lengths were tailored to be 2, 5, and 10 mm for facilitating the penetration of the epidermis/dermis, subcutaneous tissue, and deep body cavity, respectively. The mechanical strength of these needles was measured using a compression machine. The maximum tolerated compressive force was observed to be positively correlated with the base diameter but negatively related to the needle length, as the compression failure force of the needle with a length of 2 mm was greater than that of the needle with a length of 5 and 10 mm, when the needle type and base diameter was constant (**Figure** **2**A–D). This discrepancy should arise from the substantially smaller force‐bearing area near the tip of the tall needle compared to the short needle, rendering the former more susceptible to crush. This principle also accounts for the enhanced mechanical strength of the type II needle relative to the type I needle (Figure 2A–D). Consequently, type I needles readily penetrated the skin at a 2‐mm length, resulting in minimal bleeding and rapid wound healing within less than 90 min (Figure 2E). However, the type II needle was more suited for penetrating deeper tissue, albeit with a slower wound healing rate compared to type I needles, achieving full recovery within 8 days even using 10 mm‐long sampling needles (Figure 2E). Furthermore, these sampling needles maintained structural integrity (Figure 2F), and exhibited no significant reduction in mechanical strength (Figure 2G; Figure S2, Supporting Information) and mass (Figure S3, Supporting Information) for at least 3 days post‐percutaneous insertion, demonstrating their mechanical stability in vivo. In addition, the prolonged implantation of the sampling needles did not significantly restrict rats’ daily activities. These observations support the device's feasibility for sustained applications. Meanwhile, these sample needles did not induce significant immunogenic responses including visible skin irritation reactions (redness and swelling) (Figure 2E) and increased infiltration of inflammatory cells (neutrophils and macrophages) within rats’ tissues compared to the control group (Figure 2E,H; Figure S4, Supporting Information). Additionally, the resin needles possessed great cytocompatibility and hemocompatibility that are also keys for safe in vivo applications, as evidenced by their negligible cytotoxicity against endothelial (HUVEC) and fibroblast cells (NIH/3T3) (Figure S5, Supporting Information), and no observed hemolysis activity (Figure S6, Supporting Information). Together, these findings highlight the significance of tailoring the parameters of the sampling needles for adapting to diverse healthcare settings, ensuring effective tissue penetration while minimizing potential tissue damage and adverse reactions.

### 2.3 Precise and Controllable Fluid Extraction with a Simple Pressing Operation and the Microchannel Structures

**Figure 3:**
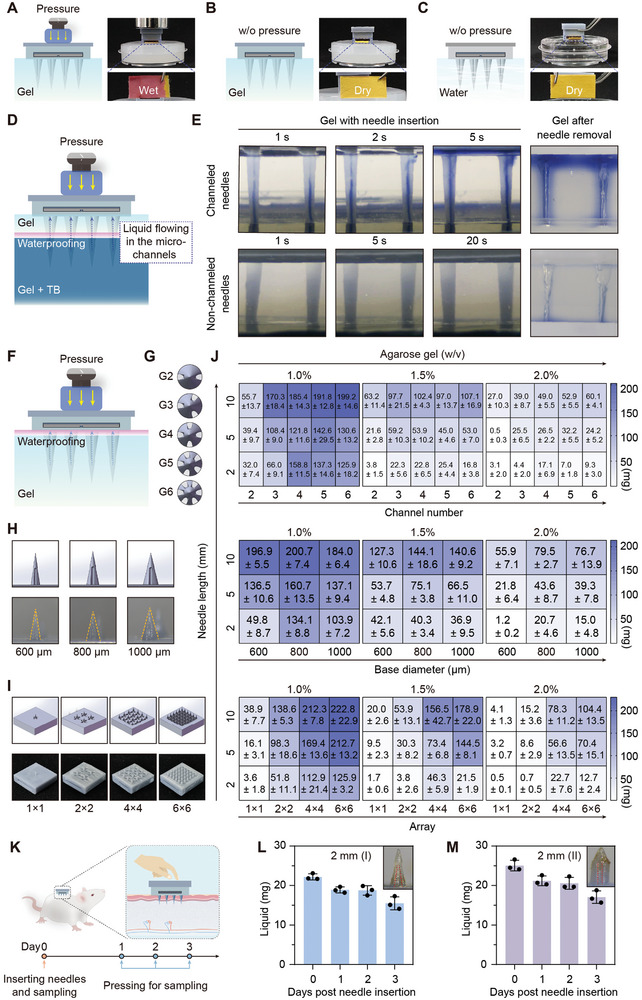
The liquid extraction capability of the MS‐MBT device with sampling needles varied by multiple external conditions and needles’ structural parameters. A–C) The schematics and images showing the liquid extraction performance (reflected by the liquid infiltration area of reaction papers) of the device with surface‐channeled needles under a pressure of 200 g weight for 60 s in agarose gel (1.5% w/v), without (w/o) pressure in agarose gel (1.5% w/v), or without (w/o) pressure in pure water. D, E) The schematic and time‐series images showing the liquid extraction performance of the device with surface‐channeled or surface‐smoothed (non‐channeled) needles under a pressure of 500 g weight in the waterproof membrane‐separated double‐layer agarose gel (1.5% w/v). TB, trypan blue. F) The schematic showing the liquid extraction of the device under pressure of 500 g weight in the waterproof membrane‐covered agarose gel. G–I) The images of the sample needles (type I) with different channel numbers (G), base diameters (H), and array patterns (I). J) The quantification of liquid extraction (mg) using the device with different sample needles (type I) in the waterproof membrane‐covered agarose gels (1.0%, 1.5%, or 2.0% w/v). Upper panel, needles possess the same base diameters (800 µm) and array pattern (4 × 4), but different channel numbers (2–6); middle panel, needles possess the same channel number (4) and array pattern (4 × 4), but different base diameters (600, 800, or 1000 µm); and lower panel, needles possess the same channel number (4) and base diameters (800 µm), but different array patterns (1 × 1 to 6 × 6). K) The schematic showing the sustained sampling process with the MS‐MBT device in vivo. L, M) The quantification of liquid extraction using the device with type I (L) or type II (M) sampling needles (2 mm in needle length, 800 µm in base diameter, 2 × 2 in array pattern) at the given time points. The insets, the images of the needles removed from rats’ dorsal tissues after sampling once daily for 3 days. The red dashed lines outline the sampling channels with minimal tissue residues. Data are presented as mean ± SD.

While various sampling devices were reported, the major challenge facing sampling is how to effectively collect samples with sufficient quality and quantity for convenient testing and analysis toward timely diagnosis.^[^ ^11^, ^13^ ^]^ Intriguingly, the liquid extraction efficiency of the MS‐MBT device highly depended on applied pressure (**Figure** **3**A; Figure S7, Supporting Information). Simply applying a pressure of 500 g weight (close to the human finger pressure under normal physiological state) quickly (≈30 s after weight applied) extracted the sufficient amount of liquid (at least 20 µL) from the tissue‐mimicking agarose gel (1.5% w/v) to infiltrate all the reaction papers in one reaction block (Figure S7 and Video S2, Supporting Information). However, when the sampling needles were inserted into the gel or even into pure water, no liquid was extracted without pressure applied (Figure 3B,C; Figure S8 and Video S2, Supporting Information), demonstrating a highly controllable sampling process that would effectively prevent undesired contamination onto reaction papers. Moreover, this highly efficient and rapid sampling was determined to be mediated by the microchannels on the needles’ surface most likely due to an enhanced capillary action,^[^ ^16^ ^]^ as the liquid extraction efficacy of the surface‐smoothed needles was markedly decreased (40–100 folds lower than channeled needles) under the same pressure (Figure 3D,E; Figure S9 and Video S3, Supplementary Video). Of note, the gels were covered with a waterproof membrane to resemble the outer skin and to ensure that the liquid flow was directed into the testing compartment through microchannels, rather than allowing liquid to seep from the compressed gel's surface into the compartment (Figure 3D,F).

To improve the sampling performance, the needles with different numbers of channels, base diameters, and arrays were optimized (Figure 3F–I). The liquid extraction efficiency of the MS‐MBT device with different needles was examined in agarose gels with low to high concentrations to simulate a spectrum of tissue hardness. To ensure an efficient sampling, we selected the 500‐g weight to apply a 60‐s pressure for the subsequent investigations. When the needle length was 10 mm, the liquid extraction efficiency was progressively improved as the number of channels increased; but the optimal sampling efficiency was achieved with 4 or 5 channels when the needle length was 2 or 5 mm (Figure 3J, upper panel; Table S1, Supporting Information). Additionally, the sampling efficiency of the needle with a base diameter of 800 µm surpassed that of the counterparts with both 600‐µm and 1000‐µm base diameters (Figure 3J, middle panel; Table S1, Supporting Information). These might be attributed to the narrowed oval inlets caused by the increase in the channel number and base diameter, consequently affecting liquid entering dynamics. Further, when the channel number and base diameter were held constant, the sampling efficiency presented a stable positive correlation with the number of arrays (Figure 3J, lower panel; Table S1, Supporting Information). A similar pattern of sampling efficiency varied by needle and array parameters was also observed for the type II needles (Figure S10 and Table S1, Supporting Information). Based on these findings, the sampling needle array with 4 channels per needle, a base diameter of 800 µm, and an array pattern of 4 × 4 was selected for subsequent in vitro experiments.

Further, to determine the potential of the sampling needles for sustained sampling in vivo, we inserted the needles into rats’ dorsal tissues, followed by sampling once daily for 3 consecutive days (Figure 3K). Although a gradual decline in sampling efficiency was observed over time, the channels remain sufficiently open to allow sample collection and interaction with test strips (Figure 3L,M; Figure S11, Supporting Information). Additionally, after removing the needles on the third‐day postinsertion, only minimal tissue residues were found within the channels (Figure 3L,M, the insets). These observations support the sustained sampling functionality of the MS‐MBT device in vivo.

### 2.4 Immediate and Persistent Multiplex Biochemical Testing with a Wireless ColorPicker

**Figure 4:**
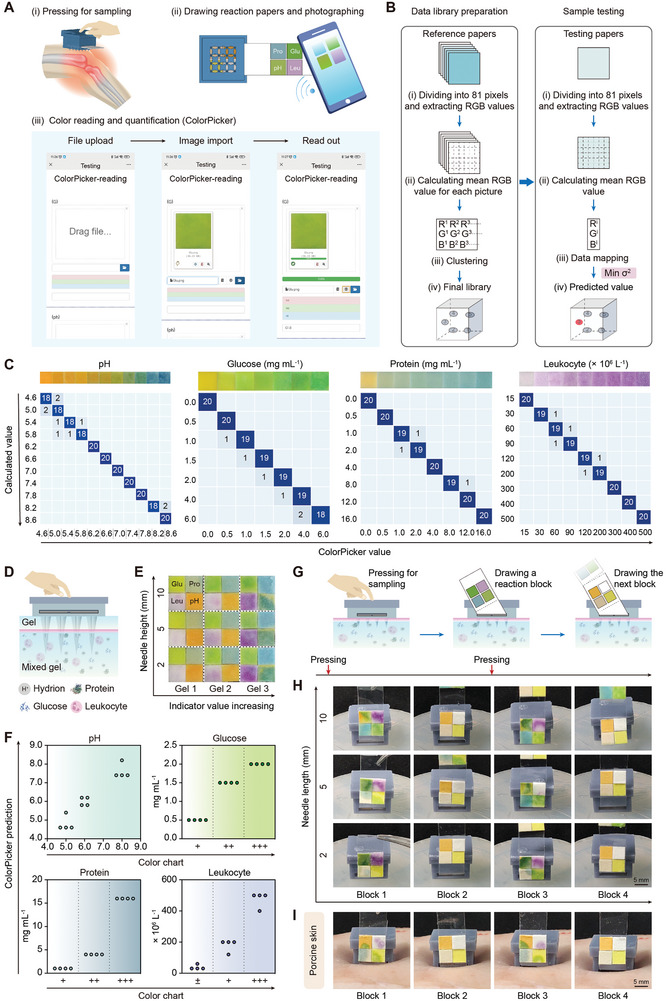
Continuous quantitative analysis of multiple biochemical indicators. A) Schematics and smartphone screen images showing the collection of deep wound samples, the acquisition of reaction paper images, and the quantification of biochemical indicators with ColorPicker. B) The flowchart and principle of quantifying the biochemical indicators by ColorPicker. Min σ^2^, Minimum variance. C) Confusion matrices visualizing the agreement between the actually calculated and ColorPicker‐obtained values or concentrations for pH, Glu, Pro, and Leu. The dark blue squares highlight the number of accurately predicted samples from the total samples (*n* = 20). D) Schematic illustration of the collection and detection of the 4 indicators with the device from a deep tissue wound‐mimic double‐layer agarose gel (1.5% w/v). E) The images of the reaction papers obtained from the device's testing compartment after the removal of the sampling needles with the given length from the gel contain the 4 mixed indicators at different values or concentrations. F) Scatter diagrams showing the agreement between the ColorPicker‐predicted and manually‐read (according to the standard color charts) results (*n* = 4). G) Schematic illustration of continuous collection and detection of the 4 indicators from the mixed gel. H, I) Images showing the colored reaction papers and their next uncolored papers drawn from the testing compartments of the devices with the given needle lengths post the removal of the weight. Upon mixing agarose gel (1.5% w/v) (H) or porcine skin (I). According to the porcine skin's thickness (≈3 mm), the device with a needle length of 5 mm was selected for sampling and detection in (I).

To demonstrate the feasibility of the MS‐MBT device in monitoring wound status, 4 dry‐chemical reaction papers that are clinically used for urinary chemical examination were selected to profile wounds at ionic (pH), small molecular (glucose, Glu), macromolecular (protein, Pro), and cellular (leukocyte, Leu) levels, respectively (**Figure** **4**A). The reaction papers are typically impregnated with chemical reagents that react with the specific analytes to generate a visible color change. The reaction principles of the indicators and the corresponding color‐changing patterns are presented in Table S2 (Supporting Information). Clinically‐used, dry‐chemical reaction paper readings are generally semi‐quantitative and rely on manual comparison with the standard color charts to obtain results.^[^ ^17^ ^]^ To improve detection precision and reliability by avoiding errors often derived from manual interpretation, a software named ColorPicker that performed an RGB color extraction and pixel‐based calculation on reaction papers was developed (see details about ColorPicker development in the Methods) and was accessible via regular smartphones for online usage, enabling rapid quantification of biochemical indicators with the help of a smartphone's camera in an effortless fashion (Figure 4A). This procedure involved photographing reaction papers, reading images, analyzing color changes, and outputting results (Figure 4A). According to the ColorPicker processing, the reaction papers yielded significantly distinct RGB color values for pH, Glu, Pro, and Leu indicators (Figure S12, Supporting Information). Moreover, the quantification of the indicators was achieved through the library preparation of reference reaction papers and the subsequent data mapping utilizing variance analysis of RGB values (see details in the Method; Figure 4B). The robust predictive capability of ColorPicker was demonstrated by the confusion matrices that revealed the accuracies achieving 90%–100% in quantifying the indicators at given values or concentrations (Figure 4C). To further assess the ColorPicker's detection stability, we conducted ten repeated tests for each indicator and calculated the relative standard deviation (RSD), with results showing an RSD of 0% across all the tests (Figure S13, Supporting Information), demonstrating the high stability of the ColorPicker‐based biochemical testing.

Next, to investigate the device's potential to simultaneously detect the 4 indicators in the samples from complex deep tissue locations, we prepared a deep tissue wound‐mimicking double‐layer agarose gel, in which the lower layer was mixed with the 4 indicators (Figure 4D). The needles with a given length were inserted into the gel followed by pressing on the top cap for sampling and indicator reactions. The color development was correlated with the indicators’ levels and was clearly exhibited in the 4 test papers obtained from the device's testing compartment after removing the sampling needles from the mixed gels (Figure 4E), suggesting that the device effectively extracts all target substances from the gels without any mutual interference across the 4 reactions in the testing compartment. The indicator levels were further quantitatively analyzed with ColorPicker software, and the obtained results presented a trend in line with the manual readings from the standard color charts that however only offer semi‐quantitative outcomes (Figure 4F). Moreover, we compared the device's results with measurements from clinical standard instruments (automated biochemical and hematology analyzers) and obtained relative errors of < 5% for Glu, < 2% for Pro, and < 8% for Leu (Table S3, Supporting Information), suggesting the reliability of the device for practical multi‐indicator detection. Additionally, we revealed the device's feasibility for continuous sampling and biochemical testing using the mixed gel (Figure 4G,H; Video S4, Supporting Information) and porcine skin tissue (Figure 4I; Video S4, Supporting Information), since the infiltration and color development of the previous reaction papers after pressing did not contaminate the subsequent papers owing to the presence of the spaced waterproof protective film (Figure 4H,I). Together, these data suggest that through the integration of ColorPicker into a smartphone, the device allows for convenient, precise, and continuous monitoring of multiple biochemical indicators within tissues, thereby facilitating swift detection of abnormalities toward improving wound management.

### 2.5 Monitoring of Deep Wounds Infected with *S. aureus* In Vivo

**Figure 5:**
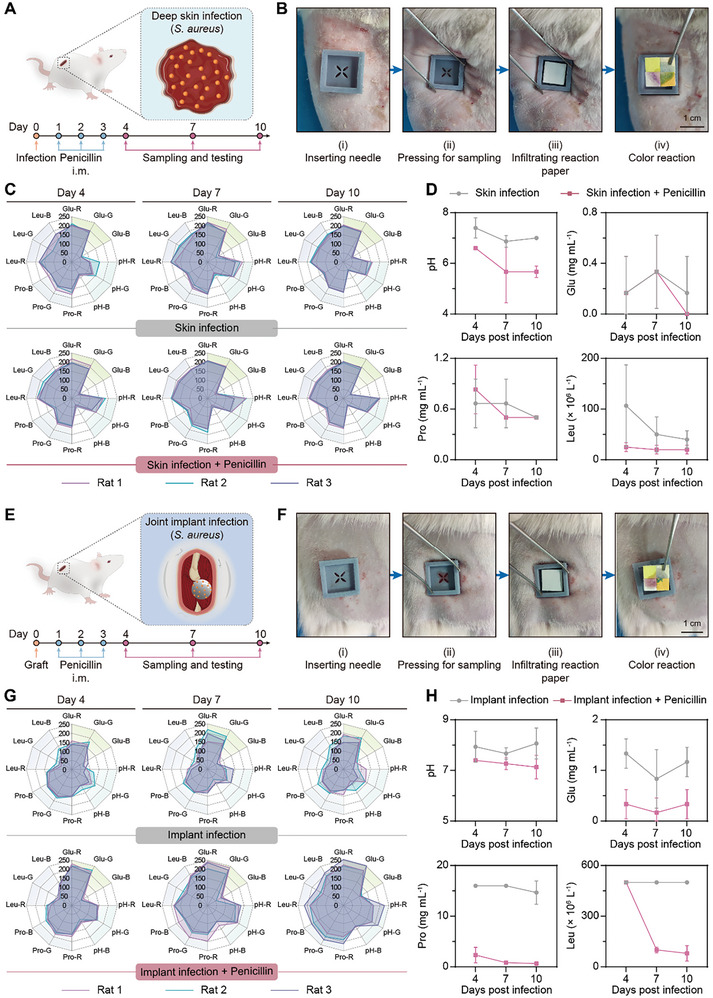
Monitoring of deep skin wound and hip joint implant‐induced wound infected with *S. aureus* in rats. A) Schematic illustration of the rat model with *S. aureus* infection in the deep skin wound, the therapeutic regimen, and the monitoring modality. B) Images showing the process of wound monitoring by local exudate sampling and testing in deep skin wound‐infected rats. C) Radial graphs showing the RGB values of the pH, Glu, Pro, and Leu indicator papers collected from the deep skin wound‐infected rats with or without penicillin treatment at the given timepoints (*n* = 3). D) The corresponding pH, Glu, Pro, and Leu levels were obtained by the ColorPicker analysis (*n* = 3). E) Schematic illustration of the rat model with *S. aureus* infection in hip joint implant, the therapeutic regimen, and the monitoring modality. F) Images showing the process of wound monitoring by exudate sampling and testing in the rats with *S. aureus* infection in the hip joint implant. G) Radial graphs showing the RGB values of the pH, Glu, Pro, and Leu indicator papers collected from the hip joint implant‐infected rats with or without penicillin treatment at the given timepoints (*n* = 3). H) The corresponding pH, Glu, Pro, and Leu values or concentrations obtained by ColorPicker analysis (*n* = 3). Data are presented as mean ± SD.

To assess the MS‐MBT device's potential for in vivo monitoring complicated deep wounds, 2 kinds of preclinical deep wound infection models were established using Sprague‐Dawley (SD) rats, i.e., *Staphylococcus aureus* (*S. aureus*) infection within deep skin location (under dermis layer) and hip joint implantation (**Figure** **5**A,E). The infected rats were randomly divided into 2 groups, one of which received a three‐day course of antibiotic treatment with penicillin (Figure 5A,E). The device was then deployed in the rat model by percutaneously inserting the needles into the wound region for exudate sampling followed by the ColorPicker's analysis on the colored reaction papers (Figure 5A,B,E,F; Video S5, Supplementary Video). Obviously, the exudate‐infiltrated reaction papers from the untreated rats with deep skin infection presented a more distinct color change for pH and Leu, when compared to those from the penicillin‐treated rats (Figure 5C; Figure S14, Supporting Information). According to the results from the ColorPicker's analysis, the penicillin treatment normalized the exudate's pH to the level of healthy skin tissue (pH 5–6)^[^ ^18^ ^]^ 7 days after the infection, accompanied with a decrease in Leu and Pro concentration, but without a significant impact on Glu levels (Figure 5D). Concurrently, the penicillin‐treated wounds exhibited complete healing across all layers of skin tissue with the absence of obvious local inflammatory exudation (Figure S15, Supporting Information). However, the untreated wounds exhibited a natural decline in both pH value and Leu concentration over time, mainly attributed to the inherent wound healing and inflammation regulating processes (Figure 5D). These findings suggest that the device effectively detects the dynamic changes of wound pH, inflammatory proteins, and cells, providing valuable information for monitoring wound infection and inflammation, healing outcomes, and therapeutic efficacy. Notably, the rats infected with *S. aureus* in deep skin revealed no significant alterations in serum inflammatory factors (TNF‐α, IL‐6, and CRP) following either penicillin treatment or throughout the observation period (Figure S16, Supporting Information). However, the untreated rats exhibited an apparent inflammatory exudation and poor wound healing compared to the penicillin‐treated ones upon opening their wounds on day ten after infection (Figure S15, Supporting Information). These findings imply the limitation of systemic serum biomarkers in faithfully reflecting the pathophysiological status of localized wound infection.

Further, we evaluated the capability of the device for monitoring surgical implantation‐related wounds with *S. aureus* infection in implanted hip joints (Figure 5E,F). The inflammatory responses derived from the hip joint implant infection were more serious than that from the aforementioned deep skin infection, since the exudate collected from the implant location generated more significant color changes on the 4 reaction papers, with the corresponding pH, Glu, Pro, and Leu levels higher than those in the exudate from deep skin infection at the same time points (Figure 5G,H; Figure S17, Supporting Information). These findings underscore the significance of multilevel biochemical examination for precisely evaluating surgical implant infection. Moreover, the penicillin treatment significantly reduced the pH, Glu, Pro, and Leu levels, whereas the 4 indicators in the exudate from the rats without anti‐inflammatory treatment maintained persistently high levels (Figure 5G,H; Figure S17, Supporting Information), indicating a failure of spontaneous wound healing. Consistently, the penicillin‐treated rats exhibited a decrease in the local purulent exudate at the surgically exposed implant wound (Figure S18, Supporting Information) and serum‐derived inflammatory factors (Figure S19, Supporting Information). These data collectively demonstrate the robust capability of the device to facilitate diagnosis and treatment for deep surgical implant infections through in situ sampling and instantly detecting multiple biochemical indicators.

### 2.6 Early Detection, Continuous Monitoring, and Inflammation Relief of Intestinal Leakage In Vivo

**Figure 6:**
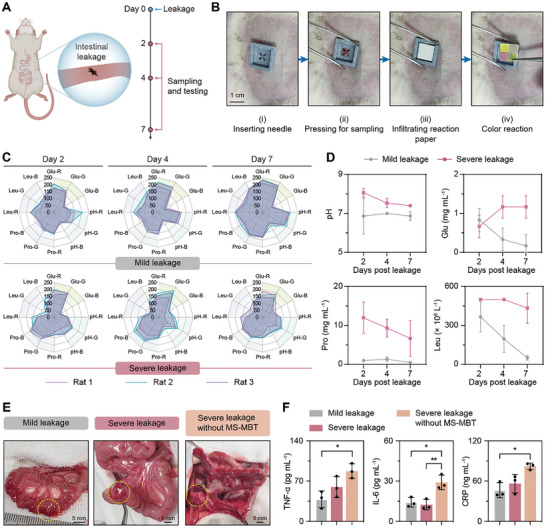
Diagnosis and monitoring of intestinal leakage, and remission of inflammatory response in rats. A) Schematic illustration of the rat model with intestinal leakage and the monitoring modality. B) Images showing the process of intestinal leakage monitoring by local leakage liquid sampling and testing. C) Radial graphs showing the RGB values of the pH, Glu, Pro, and Leu indicator papers collected from the rats with mild or severe intestinal leakage at the given timepoints (*n* = 3). D) The corresponding pH, Glu, Pro, and Leu values or concentrations were obtained by ColorPicker analysis (*n* = 3). E) The intestinal images acquired on day 7 post‐surgery. The yellow dotted circles highlight the surgical leakage regions. F) Serum inflammatory cytokine levels in the rats with mild or severe intestinal leakage with or without MS‐MBT device treatment on day 7 post‐surgery (*n* = 3). Data are presented as mean ± SD. *p < 0.05, **p < *0.01*; one‐way ANOVA.

To determine the potential of the MS‐MBT device in diagnosis and monitoring postoperative intestinal leakage, the transverse incisions were created in rats’ intestines (**Figure** **6**A) that harbor abundant microorganisms, rendering surgical incisions particularly susceptible to bacterial infection.^[^ ^19^ ^]^ These incisions were subsequently monitored using the device coupled with ColorPicker, which effectively detected intestinal leakage from both long and short incisions on the second day after surgery, as the liquid collected from the incision location rapidly infiltrated the reaction papers followed by a prompt color reaction (Figure 6B,C; Figure S20 and Video S6, Supplementary Video). Further, the ColorPicker readings revealed higher pH, Glu, Pro, and Leu levels in the severe leakage liquid concomitantly with aggravating local intestinal inflammation (redness and swelling) and systemic inflammatory factor levels, compared to those in the mild leakage liquid throughout the observation period (Figure 6D–F; Figure S21, Supporting Information). Unexpectedly, the treatment with MS‐MBT device alleviated the local and systemic inflammatory responses induced by severe intestinal leakage, most likely due to the active drainage of infectious and inflammatory leaked fluid, as more pronounced intestinal swelling, adhesion, and obstruction, along with elevated levels of serum inflammatory factors observed in the severe intestinal leakage‐stricken rats without MS‐MBT device's deployment (Figure 6E,F). These observations suggest the potential utility of the device not only in early detection, severity evaluation, and continuous monitoring of intestinal leakage but also in reducing inflammatory responses, thereby possibly preventing the progression of intestinal leakage through both diagnostic and therapeutic intervention.

### 2.7 Accurate Identification of Pathogenic Bacteria In Vitro and In Vivo

**Figure 7:**
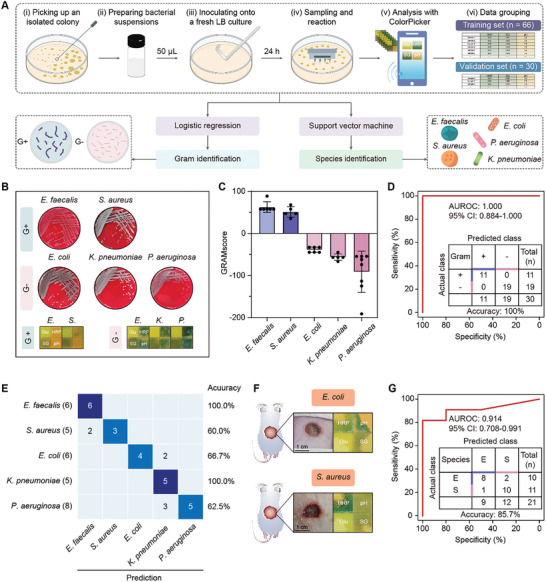
Bacterial identification with MB‐MBT device and machine learning. A) The schematic showing the experimental design for bacterial identification. B) The images of different bacterial colonies on the blood agar plate and the reaction papers infiltrated by the bacterial culture liquid collected by the device. C) The GREAsores for different bacteria are calculated by the logistic regression model. D) The ROC curve (red), AUROC value, and confusion matrix on differentiating G+ and G‐ bacteria. E) The confusion matrix showing the accuracies of bacterial classification. F) The images showing the *E. coli*‐ and *S. aureus*‐infected wounds in rats and the corresponding reaction papers infiltrated by the wound exudate collected by the device. G) The ROC curve (red), AUROC value, and confusion matrix on differentiating *E. coli*‐ and *S. aureus‐*infection in rats.

Early and accurately identifying the pathogens involved in wound infection is critical to informed clinical decisions, allowing clinicians to initiate appropriate and targeted antibiotic therapy for preventing abuse and overdose of broad‐spectrum antibiotics and minimizing the risk of antibiotic resistance. To this end, we set to investigate whether the device integrating effective sampling and multiplex biochemical testing could facilitate the identification of pathogens, including distinguishing between Gram‐positive (G+) and Gram‐negative (G‐) bacteria, as well as classifying multiple pathogenic bacteria (**Figure** **7**A). Five prevalent pathogenic bacteria known to participate in various wound infections and impede healing, namely *Enterococcus faecalis* (*E. faecalis*, G+), *S. aureus* (G+), *Escherichia coli* (*E.coli*, G‐), *Klebsiella pneumoniae* (*K. pneumoniae*, G‐), and *Pseudomonas aeruginosa* (*P. aeruginosa*, G‐), were individually inoculated onto lysogeny broth (LB) nutrient agar in accordance with a standardized protocol, followed by insertion of the device's needles for culture sampling and subsequent biochemical analysis with ColorPicker (Figure 7A,B). Based on manual readings, 3 biochemical indicators, i.e., pH, HRP, and SG, displayed distinct color changes across the 5 bacterial species and thus were selected for developing bacterial identification models (Figure 7B; Figure S22, Supporting Information). Subsequently, the quantitative analysis of these indicators was performed using ColorPicker, with accuracies ranging from 85% to 100% (Figure S23, Supporting Information). The obtained biochemical data from 96 qualified bacterial samples were eventually collected and randomly divided into a training set (*n* = 66) and a validation set (*n* = 30) (Figure 7A). The Gram identification model was established using logistic regression that provided a formula (1480.27 × SG – 39.05 × HRP–30.19 × pH–1299.22) for calculating GRAMscore (Figure 7A,C). Impressively, from the receiver operating characteristic (ROC) curve, the model achieved an area under ROC curve (AUROC) value of 1.000 (95% CI: 0.884–1.000) with an accuracy of 100% (95%CI: 88.7%–100%) in the validation set (Figure 7D), demonstrating the remarkable efficacy of the model in distinguishing between G+ and G‐ bacteria. Furthermore, by employing support vector machine (SVM) algorithm, these 3 biochemical indicators could classify the 5 bacterial species with an overall accuracy of 76.7% (95%CI: 59.1%–88.2%) and an accuracy of 100% for distinguishing *E. faecalis* and *K. pneumoniae* from the others (Figure 7A,E). Finally, the device's potential to identify bacterial species by detecting these biochemical indicators was validated in the rat skin wound infection model using logistic regression analysis (GRAMscore = 7.79 × HRP + 0.82 × pH + 450.53 × SG–465.60), and yielded an AUROC of 0.914 (95% CI: 0.708–0.991) and an accuracy of 85.7% (95%CI: 65.36%‐95.02%) for differentiation between *S. aureus* and *E. coli* (Figure 7F,G). Notably, the AUROC value for the combination of pH, HRP, and SG was higher than that for any individual indicators in identifying these 2 bacteria (Figure 7G; Figure S24, Supporting Information), underscoring the significance of employing multiple biomarkers for bacterial identification. Collectively, these findings demonstrate the feasibility of utilizing MS‐MBT device enhanced with machine learning for not only distinguishing Gram characteristics but also classifying specific bacterial species, highlighting its versatility and reliability in wound management.

## 3 Discussion

Accurate and continuous monitoring of deep wounds is critical to the early detection and elimination of any obstacles to wound healing. However, this operation poses considerable challenges mainly owing to the difficulties in external observation and assessment. Here, we proposed a miniaturized semi‐implantable device capable of collecting fluid samples from deep wounds and performing continuous biochemical testing using a smartphone. Compared to cumbersome imaging diagnoses or clinical symptoms, signs, or blood examinations, the collection of samples directly from wound sites for biochemical analysis proves to be a more convenient and efficacious approach for obtaining accurate information regarding the actual wound conditions. Additionally, sampling needles’ structure and dimensional parameters are highly tunable, offering extensive possibilities for diverse applications across various injured tissues. This study revealed that significant differences among distinct treatment groups in the biochemical indicators of fluid extracted from wound sites (including deep skin, hip joint, and intestinal wounds) generally appeared much earlier than differences observed in inflammatory factors from peripheral blood. These findings demonstrate the temporal advantage of employing indicators directly obtained from the wound site, offering timely warnings and treatment initiation.

The selection of informative indicators is also pivotal in accurately and comprehensively indicating the wound status. The emerging intelligent platforms for in situ monitoring wounds primarily focus on detecting markers at small molecule levels. For instance, elevated pH, uric acid, and lactic acid generally indicate wound infection, inflammation, and impaired healing.^[^ ^20^ ^]^ However, the available small molecule markers indicative of wound status are rather limited. These molecules are highly susceptible to interference from external and internal milieu as well as daily dietary constituents. Consequently, the accuracy and reliability of wound diagnosis may be reduced when exclusively relying on a single or a few small molecule markers. For example, decreased glucose levels in wound exudate may indicate bacterial activity;^[^ ^6b^ ^]^ but glucose levels in diabetic wounds exhibit a strong correlation with blood sugar levels and can be easily influenced by dietary intake.^[^ ^6^, ^11^ ^]^ Our study revealed increased glucose in the liquid from *S. aureus*‐infected wounds without antibiotic treatment and severe intestinal leakage, suggesting that the glucose levels in the wound environment may be varied by bacterial species present or be influenced by other metabolic cell types, and even systemic hormonal regulation. Comparatively, protein molecules and cell composition within wound liquid may provide more stable and valuable diagnostic information for wound assessment, since wound healing or deterioration involves numerous complex factors, including abundant protein components (cytokines, proteases, immunoglobulins, albumin, etc.) and various crucial cells (inflammatory cells, epithelial cells, endothelial cells, fibroblasts, etc.).^[^ ^15^, ^21^ ^]^ We observed significant variations in total protein and Leu concentrations within wound liquid among different treatment groups, highlighting the importance of these 2 types of indicators for wound diagnosis. Further identification of specific protein molecules and cell subtypes would contribute to more precise stratification of wound status and prognostic prediction.

Reducing the detection cost of wound indicators and minimizing potential harm to health is critical to supporting successful clinical translation. Although the advanced electronic sensors allow for simultaneous and rapid detection of multiple diagnostic biochemical molecules and physical signals for wound status, their utilization in clinic settings may face challenges due to a necessity for incorporating a variety of complex electronic components with high fabrication costs and reduced biocompatibility.^[^ ^20^, ^22^ ^]^ Additionally, electronic platforms might be disposable or require frequent replacements, thus substantially increasing medical costs, particularly in the case of serious chronic wounds that need prolonged and continuous management. Here, we employed the cost‐effective dry‐chemical reaction papers (also termed reagent strips), commonly utilized for chemical examination of urine in the clinic. Their potential applicability for wound liquid testing and diagnosis was revealed in this study. To achieve convenient continuous monitoring of wound status, we integrated the sampling and testing processes into a single, portable device, but spatially separating the sampling module from the testing module that was loaded with the continuously withdrawable reaction strips. Therefore, while enabling continuous wound monitoring, the MS‐MBT device ensures that chemical reagents do not directly contact wound tissues to prevent adverse reactions. Further, we have verified the cytocompatibility, hemocompatibility, and histocompatibility of sampling needles that directly contact wounds; however, rigorous long‐term safety evaluation in large animal models remains necessary.

To eliminate result errors introduced by manual readings, we developed software based on color image processing and statistical analysis of RGB values to obtain quantitative biochemical results, accessible through a smartphone. Despite its simplicity and user‐friendliness, several limitations should be acknowledged. First, the reliance on smartphone photography for color picking and analysis introduces potential interference from varying phone models and shooting conditions (such as light and shooting distance). To mitigate this limitation, we corrected the color values with a standard white color to reinforce the result reliability. Additionally, although we can precisely capture uncontaminated and evenly‐colored image areas for upload and analysis, the incidence of severe wound bleeding would still impact the accuracy of certain indicators (such as HRP owing to heme's peroxidase‐like activity). Thus, the software should be optimized by introducing reference color blocks coupled with sophisticated machine learning and deep learning algorithms for data calibration. Second, the values or concentrations of biochemical indicators are obtained by mapping the color values to the predetermined reference data, resulting in insufficient accuracy. In the follow‐up, a linear relationship between color value and indicator level would be established to achieve higher accuracy. Third, we verified the correlation of the indicators (pH, Glu, Pro, and Leu) with infected wound treatment outcomes and the severity of intestinal leakage. Still, for future advancements, a prospective dataset with a larger sample size and additional diagnostic indicators is required to develop standardized models for precisely evaluating the severity of diverse wounds and predicting healing outcomes.

The identification of pathogens is crucial in wound management, facilitating the initiation of targeted antibiotic therapy to mitigate the abuse of broad‐spectrum antibiotics and prevent the emergence of antibiotic resistance. However, this aspect is often overlooked owing to the traditional requirement of time‐consuming in vitro bacterial culture with a series of biochemical tests, or more advanced but expensive techniques, such as mass spectrometry and high‐throughput sequencing. We here identified 3 key biochemical indicators that were collected and reliably read by MS‐MBT device with ColorPicker for both in vitro and in vivo identification of common pathogenic bacteria. This platform would be helpful for promptly identifying pathogens during emergencies and monitoring wound status, particularly in challenging environments, such as wilderness settings or resource‐limited regions. We noticed that bacteria‐induced biochemical changes were susceptible to environmental influences. For instance, variations in pH, SG, or HRP levels were observed among *S. aureus* colonies in vitro culture, in deep and narrow rat wounds, and in opened rat wounds. Thus, more types of infected wound models should be developed to tailor diagnostic protocols. It is also worth noting that the mere presence of pathogens in wound regions does not necessarily indicate acute infection and inflammation. The manifestation of these conditions typically requires a substantial bacterial proliferation generating abundant toxins, as well as the deficient body immune responses failing to autonomously eliminate pathogens. This further underscores the significance of multiplexed monitoring for a comprehensive assessment of wound conditions to improve diagnostic and therapeutic strategies.

## 4 Conclusion

In summary, we developed an MS‐MBT device capable of continuously monitoring deep infected wound status through the integration of in situ sampling and immediate biochemical examination. This highly integrative system would be a valuable supplement to routine wound monitoring and postoperative management strategies, given its potential for self‐administration outside of hospital settings. Meanwhile, the device can be readily tailored with diverse structural parameters for sampling needles, along with flexible configuration of multiple reaction papers for diagnostic indicators, thus offering ample opportunities for versatile designs demanded by different wound types.

Compared to current clinical and microneedle array‐based wound monitoring approaches (Table S4, Supporting Information), the MS‐MBT portable device offers benefits, particularly in: 1) the operationally convenient fabrication process and cost‐effective materials; 2) available continuous monitoring of wound biomarkers by integrated yet spatially separated (interference‐free) sampling and detection modules; 3) effective and effortless deep sampling with well‐designed and highly tunable needles; and 4) rapid, convenient, and accurate multiplex biochemical testing by combining low‐cost dry chemical test strips with smartphone‐accessible image processing software. Thus, our approach supports potential home‐based use and remote medical guidance, reducing the need for frequent clinical visits. Future developments would aim to improve material flexibility for better accommodating wound monitoring during motion conditions, incorporate additional physical indicators (such as temperature, humidity, and impedance) and therapeutic agents for more comprehensive and closed‐loop wound management, and upgrade the image processing software with advanced machine learning algorithms for more precise quantitative analysis.

## 5 Experimental Section

### Preparation and Characterization of MS‐MBT Device

The main components of the MS‐MBT device, including the top cap (18 × 18 mm) and testing compartment (16 × 16 × 10 mm) with sampling needles (tunable length, base diameter, and array) were designed in Solidworks 2021 and printed using an X190 3D printer (Xiaoyanger, China) with UV‐curable resin (Anycubic Technology, China) as the printing material. The printing parameters were set as follows: exposure time, 5 s; lifting distance, 5 mm; lifting speed, 65 mm min^−1^; bottom exposure time, 20 s; layer height, 10 µm; return speed, 150 mm min^−1^. Two stainless‐steel springs (10 mm in length, 0.2 mm in wire diameter, and 3 mm in outer diameter; Tengluo Hardware, China) were glued inside the top cap to help reaction papers to be fixed and continuously drawn. Dry‐chemical reaction paper strips (DIRUI, China) were cut into several squares (5 × 5 mm); and 4 reaction paper squares for detecting specific biochemical indicators were combined into a 2 × 2 reaction block and pasted onto a waterproof strip (110 × 11 × 70 µm), with a gap of 11 mm maintained between each 2 reaction blocks to prevent cross‐contamination. The waterproof strip was then folded with the reaction paper side facing down and placed into the testing compartment, followed by coverage with the spring cap.

### Characterization of MS‐MBT Device

Structural characterization of the device and its components was performed by an MZ62‐type microscope (MingMei, China) and a scanning electron microscope (TESCAN VEGA3, China). The mechanical strength of different sampling needles was tested with a microcomputer‐controlled electronic universal testing machine (CMT6001, China) according to our previously reported method.^[^ ^23^ ^]^ The capabilities of different sampling needles to penetrate through skin tissue, as well as the subsequent recovery of skin wounds and immunogenic responses, were investigated on SD rats (6–8 weeks old, purchased from and raised in the Laboratory Animal Center, Huazhong University of Science and Technology, Wuhan, China) through photography, H&E staining, and immunofluorescent staining (Antibodies: MPO, ab208670; F4/80, ab300421).

### Sampling Performance Evaluation

The sampling performance of MS‐MBT device was tested in 1) different tissue‐mimic agarose gels, including mono‐layer agarose gels (1.5% w/v; 15 mm in thickness), waterproof membrane‐separated double‐layer agarose gel (1.5% w/v; upper layer, 3 mm in thickness; lower layer, 10 mm in thickness, containing 0.6 mg mL^−1^ trypan blue), and waterproof membrane‐covered mono‐layer agarose gel (1.0%, 1.5%, or 2.0% w/v), 2) rats whose dorsal tissues were pre‐injected with saline (500 µL), and 3) porcine skins obtained fresh from a local slaughterhouse (Wuhan, China) and cleaned with cold running water. Type I or type II sampling needles with specified needle lengths, base diameters, and array patterns were inserted into agarose gels under a pressure of 100, 200, 500 g weight for a given time or finger pressure. The amount of liquid extracted by sampling needles was the weight difference of filter or test paper in the device's testing compartment before and after pressing measured by an electronic balance. Agarose and trypan blue were supplied by Sinopharm Chemical Reagent (China).

### ColorPicker Development

We developed an online tool (ColorPicker, http://web.ypush.tech:8088/tk/api/pape/index2) for translating color images into concentrations or values of specific biochemical indicators. RGB values of reaction papers were acquired based on an open‐source package‐java. imageio.ImageIO. To reduce bias caused by uneven coloring, the image processing software was improved. Specifically, a reaction paper image obtained by smartphone photography was divided into 100 grids (10 × 10); and the RGB values of the 81 intersecting pixel points were extracted. The average of these 81 RGB values served as the standard color value for this reaction paper. Standard solutions containing specific biochemical indicators at graduated concentrations (or values) were prepared and reacted with corresponding reaction papers, and the successfully colored reaction papers were uploaded to the software for RGB color reading and writing. Ten reaction papers were uploaded for each concentration (or value); and the RGB mean values were calculated separately using the following formula, which serves as the reference color value for a specific indicator at the respective concentration or value.

(1) $$R_{r}=\sum R/n,\;G_{r}=\sum G/n,\;B_{r}=\sum B/n$$

∑*R*, ∑*G*, and ∑*B* represented the sums of the standard R, G, and B values from the ten (*n*) reaction papers, respectively. By employing the aforementioned methods, the reference color values for pH, Glu, Pro, Leu, HRP, and SG at the given concentrations (or values) were obtained, following clustering analysis to establish the ultimate reference data library. Citric acid hydrate, Na_2_HPO_4_·12H_2_O, HCl, tris‐base (for preparing standard solution with different pH), glucose, bull serum albumin (for preparing standard protein solution), and NaCl (for preparing standard solution with different SG) were acquired from Sinopharm Chemical Reagent (China). HRP was provided by Aladdin Biochemical Technology (China). Leu was collected from the peripheral blood.

### Quantitative Analysis, Performance Evaluation, and Optimization

Quantitative results for biochemical indicators were obtained by mapping standard color values of testing reaction papers to the reference data library through variance analysis. To evaluate the accuracy of this method, solutions containing biochemical indicators at graduated concentrations (or values) were newly configured and reacted with corresponding reaction papers. The standard color values of the reaction papers were acquired using the same method as mentioned above. For each concentration (or value), 20 reaction papers were uploaded and processed in the software. Subsequently, the variance (σ^2^) between the standard color values of testing reaction papers and the reference color values was calculated based on the following formula:

(2) $$\sigma^{2}={\left(R-R_{r}\right)}^{2}+{\left(G-G_{r}\right)}^{2}+{\left(B-B_{r}\right)}^{2}$$

The concentration (or value) corresponding to the minimum variance was determined as the final reading of the testing reaction paper. The consistency between ColorPicker‐predicted values and actual values was visualized using confusion matrixes. To mitigate the influence of interfering factors such as phone model, lighting conditions, and shooting distance, the standard color values of a reaction paper were corrected using the standard RGB color of the white region in the corresponding reaction paper.

### In Vivo Wound Monitoring

Three preclinical wound models were established in SD rats (6–8 weeks old, purchased from and raised in the Laboratory Animal Center, Huazhong University of Science and Technology, Wuhan, China) to study the potential of MS‐MBT devices for monitoring diverse wounds. 1) Infected deep skin wound: a surgical incision with a length of 5 mm was made on the rats’ dorsal skin; and 200 µL *S. aureus* (ATCC 25 923) suspension (0.5 McFarland, ≈1 × 10^8^ CFU mL^−1^) was injected under dermis layer along the incision, followed by incision suture. 2) Infected surgical implant wound: rats’ hip skin and muscle were surgically incised to expose the hip joint, where a stainless‐steel screw (5.6 mm in head diameter, 3.5 mm in thread diameter, and 8.3 mm in total length) pretreated with *S. aureus* suspension (0.5 McFarland, ≈1 × 10^6^ CFU mL^−1^) for 24 h was implanted to simulate an artificial joint implantation surgery. 3) Intestinal leakage: mild (piercing the intestinal wall with a 26 G syringe needle) or severe (surgically cutting off 1/3 of the small intestine's circumference) intestinal leakages were created in rats’ small intestine ≈20 cm away from the ileocolic part, followed by fixing the intestine through suspending the intestinal wall with surgical sutures to the right abdominal wall, subsequently closing the abdome.^[^ ^24^ ^]^ Rats infected with *S. aureus* in deep skin or hip joint implant were randomly divided into 2 groups, one of which was treated with penicillin (8 × 10^5^ units kg^−1^, i.m., once a day, 3 times in total; Gelian Biotechnology, China). Wound sampling and testing were performed with MS‐MBT device (type II needles, 2 mm (for deep skin wounds), 5 mm (for hip joint implant wounds) or 10 mm (for intestinal leakage) in length, 800 µm in base diameter, and 1 × 1 in array pattern; sterilized with a 12 h‐alcohol soak and a 12 h‐UV irradiation) and ColorPicker at given time points. Meanwhile, peripheral blood was collected from rats’ tail vessels for isolating serum and detecting inflammatory factors (TNF‐α, IL‐6, and CRP) using ELISA assay kits (Ruixin biotech, China). To ensure sufficient wound fluid was extracted, all deep skin and hip joint implant wounds were washed with sterilized saline (30 µL) before sampling and testing. In addition, successfully colored areas in reaction papers were deliberately intercepted and uploaded to the software for image analysis.

### In Vitro Bacterial Identification

Bacterial suspensions (0.5 McFarland, ≈1 × 10^8^ CFU mL^−1^) of *E. faecalis* (ATCC 29 212), *S. aureus*, *E. coli* (ATCC 25 922), *K. pneumoniae* (ATCC 700 603), and *P. aeruginosa* (ATCC 27 853) were prepared and inoculated separately in LB nutrient agar containing glucose (1 mg mL^−1^) with inoculating loop, followed by incubation at 37 °C for 24 h. MS‐MBT device's needles were inserted into the LB nutrient agar to extract bacterial culture liquid that was subsequently reacted with the reaction papers (for indicating pH, SG, HRP, and Glu) in the device's testing compartment. Successfully colored reaction papers were then analyzed with ColorPicker.

To establish models for the identification of Gram types and bacterial species, 96 samples (*n* = 20 for *E. faecalis*; *n* = 17 for *S. aureus*; *n* = 20 for *E. coli*; *n* = 19 for *K. pneumoniae*; *n* = 20 for *P. aeruginosa*) were randomly divided into training set (*n* = 66) and validation set (*n* = 30). Logistic regression analysis was performed on the training set using 3 indicators (SG, HRP, and pH) to construct a binary classification model for distinguishing G+ and G‐ bacteria. SVM algorithm with linear kernel was used to build a multi‐classification model to classify 5 bacterial species by these 3 biochemical indicators in R language with the e1071 package (version 1.7‐14). The performance of both models was evaluated using the samples in the training set or validation set, and the ROC curves were generated using R package pROC (version 1.18.4).

### In Vivo Identification of Different Bacterial Infections

To evaluate the in vivo discriminatory ability of 3 indicators (SG, HRP, and pH) collected by MS‐MBT device for different bacterial infections, *E. coli‐* or *S. aureus‐*infected wound models were constructed on rat's dorsal skin. Specifically, a piece of dorsal skin with a diameter of 1 cm and a thickness of 2 mm was removed from the rat's dorsum using a hole puncher; and 200 µL of bacterial suspension (0.5 McFarland, ≈1 × 10^8^ CFU mL^−1^) was dripped onto the wound region that was then covered with a sterile dressing. After 2 days, wound liquid was extracted with the device's needles (2 mm in length, 800 µm in base diameter, and 1 × 1 in array pattern) for biochemical examination with ColorPicker. 21 samples (*n* = 11 for *S. aureus*‐infected wound; *n* = 10 for *E. coli*‐infected wound) were collected. Logistic regression analysis was performed on the collected samples using combined SG, HRP, and pH to construct a binary classification model for distinguishing bacterial species in vivo. The ROC curves were generated using the R package pROC (version 1.18.4).

All the experiments received ethical approval from the institutional animal care and use committee of Huazhong University of Science and Technology, Wuhan, China ([2023] IACUC Number: 3742).

### Statistical Analysis

Data were presented as mean ± standard deviation (SD) and statistically analyzed by Student's *t*‐tests (2 independent data sets) or one‐way ANOVA with post hoc tests (more than 2 independent data sets). Statistical significances were indicated by ^*^p < 0.05, ^**^p < 0.01, ^***^p < *0.001*, and N.S. (not significant). Confidence intervals (CI) for AUROC were calculated with AUROC ± 1.96 × standard error (SE, obtained by DeLong's method).

## Conflict of Interest

The authors declare no conflict of interest.

## Supporting information



Supporting Information

Supplementary Video 1

Supplementary Video 2

Supplementary Video 3

Supplementary Video 4

Supplementary Video 5

Supplementary Video 6

## Data Availability

The data that support the findings of this study are available from the corresponding author upon reasonable request.
