# Supplementary material for: A Smart Semi‐Implantable Device Integrating Microchannel‐Enhanced Sampling and Multiplex Biochemical Testing for Deep Wound Monitoring and Pathogen Identification
Source: Adv Sci (Weinh). 2024 Dec 31;12(8):2407868. doi: 10.1002/advs.202407868 (PMC11848630; doi:10.1002/advs.202407868)
Supplement: Supplementary file 1 — Supporting Information [file ADVS-12-2407868-s004.docx]

Supporting Information

**A Smart** **Semi-implantable Device** **Integrating** **Microchannel-enhanced Sampling and** **Multiplex Biochemical Testing for Deep Wound Monitoring and Pathogen Identification**

*Qilin Li, Chunyu Wei, Luming Xu, Jiao Zhang, Yuyu Li, Xiaohuan Lu, Rengui Xu, Honglian Guo, Peng Cao, Chenke Ouyang, Jiarong Xu, Wei Chen^*^, Zheng Wang^*^, Lin Wang****^*^***

Q. Li, C. Wei, L. Xu, J. Zhang, Y. Li, R. Xu, H. Guo, P, Cao, C. Ouyang, W. Chen, L. Wang

Department of Clinical Laboratory, Union Hospital, Tongji Medical College, Huazhong University of Science and Technology, Wuhan 430022, China

E-mail: lin_wang@hust.edu.cn; weichen86@hust.edu.cn

Q. Li, C. Wei, L. Xu, Y. Li, X. Lu, R. Xu, H. Guo, P, Cao, C. Ouyang, Z. Wang, L. Wang

Hubei Key Laboratory of Regenerative Medicine and Multi-disciplinary Translational Research, Hubei Provincial Engineering Research Center of Clinical Laboratory and Active Health Smart Equipment, Research Center for Tissue Engineering and Regenerative Medicine, Union Hospital, Tongji Medical College, Huazhong University of Science and Technology, Wuhan 430022, China.

E-mail: zhengwang@hust.edu.cn;

X. Lu, Z. Wang

Department of Gastrointestinal Surgery, Union Hospital, Tongji Medical College, Huazhong University of Science and Technology, Wuhan 430022, China

J. Xu, W. Chen

Department of Pharmacology, School of Basic Medicine, State Key Laboratory for Diagnosis and Treatment of Severe Zoonotic Infectious Diseases, Tongji-Rongcheng Center for Biomedicine, Tongji Medical College, Huazhong University of Science and Technology, Hubei Key Laboratory for Drug Target Research and Pharmacodynamic Evaluation, Huazhong University of Science and Technology, Wuhan 430030, China

**Contents**

Supporting methods

Figure S1. Composition of the serial reaction paper strip.

Figure S2. Assessment of the mechanical stability *in vivo*.

Figure S3. Assessment of the needle mass loss.

Figure S4. Assessment of the inflammatory response.

Figure S5. Assessment of device materials’ cytocompatibility.

Figure S6. The hemolysis activity analysis.

Figure S7. Sampling efficiency varied by pressure, needle length, and agarose mass concentration.

Figure S8. Liquid extraction performance under different conditions.

Figure S9. Liquid extraction performance of the device with surface-smoothed needles.

Figure S10. Quantification of liquid extraction using the device with type II sampling needles.

Figure S11. The sustained sampling process with the MS-MBT device *in vivo*.

Figure S12. Radial graphs of the RGB values of the biochemical reaction papers obtained by ColorPicker readings.

Figure S13. Assessment of the detection stability.

Figure S14. Real-time monitoring of *S. aureus*-infected deep skin wound in rats.

Figure S15. The wound images acquired on day ten post-infection.

Figure S16. Monitoring of serum inflammatory cytokines from the rats with *S. aureus*-infected skin wound.

Figure S17. Real-time monitoring of hip joint implant infected with *S. aureus* in rats.

Figure S18. The wound images acquired on day ten post hip joint implant infection.

Figure S19. Monitoring of serum inflammatory cytokines from the rats with hip joint implant.

Figure S20. Real-time monitoring of intestinal leakage in rats.

Figure S21. Monitoring of serum inflammatory cytokines from the rats bearing mild or severe intestinal leakage.

Figure S22. Screening of valuable biochemical indicators for bacterial identification.

Figure S23. ColorPicker analysis on the SG and HRP indicator papers.

Figure S24. The ROC curves and AUROC values on differentiating *E. coli-* and *S. aureus* infection in rats with the individual indicators.

Table S1. The quantification of liquid extraction using the device with different sample needles in the waterproof membrane-covered agarose gels.

Table S2. The reaction principles and corresponding color-changing patterns.

Table S3. Comparison of measured values between the MS-MBT device and clinically standardized quantitative methods.

Table S4. Comparisons of the wound monitoring approaches based on our MS-MBT device and other microneedle array devices.

Supporting video captions**Supporting methods**

**Cytocompatibility evaluation**

HUVEC (human umbilical vein endothelial cells) or NIH/3T3 (mouse embryonic fibroblast cell line) cells (3 × 10^5^ cells per well) were seeded in 12-well plates and cultured with Dulbecco’s Modified Eagle Medium (DMEM) containing 10% fetal bovine serum (FBS) in a humidified incubator with 5% CO_2_ at 37 ℃. Resin sampling needles (type II, 10 mm in height, 800 μm) sterilized by 75% ethyl alcohol and UV light were added into culture plates (two needles per plate) for co-incubation with HUVEC or NIH/3T3 cells. After twelve hours, HUVEC or NIH/3T3 cells were collected and stained with a Live/Dead Kit (Beyotime, China) according to the manufacturer’s introduction. Green Calcein AM and red propidium iodide (PI) were used to label live and dead cells, respectively. The stained cells were observed and photographed using a fluorescence microscope (OlympusIX73, Japan).

**Hemolysis assay**

Peripheral blood-derived red blood cells (RBCs) were washed with PBS for several times and resuspended in PBS with a concentration of 2% (v/v). Resin sampling needles (type II, 2 mm in height, 800 μm; 1, 2, 4, and 16 in number) were added to RBCs suspension and allowed to stand for two hours. RBCs suspension was then centrifuged at 3000 rpm for 5 minutes. The supernatants’ absorbance at 545 nm was determined by a microplate reader (Infinite F50, Tecan, Switzerland). RBC lysis buffer (Simgen, China) and PBS served as positive and negative controls, respectively.

**Supporting figures**


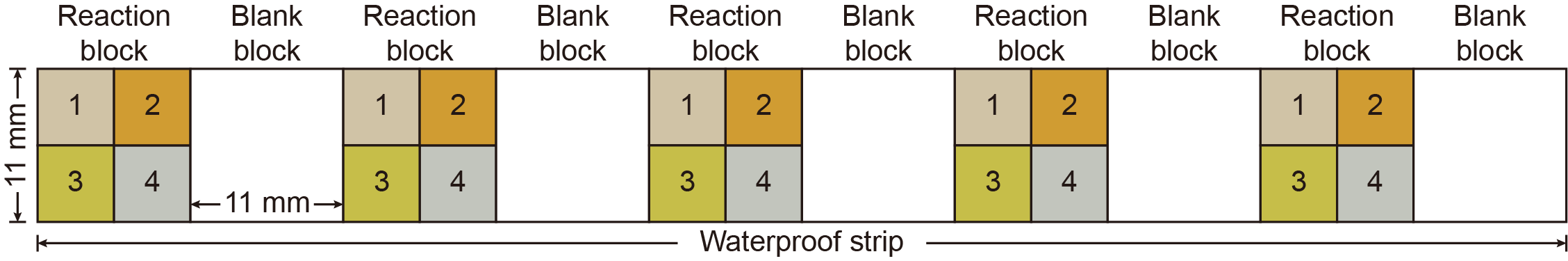


**Figure S1. Composition of the serial reaction paper strip.** Four individual reaction papers were affixed onto the waterproof strip to constitute a reaction block separated from the next reaction block by a blank block to prevent sample contamination.

**
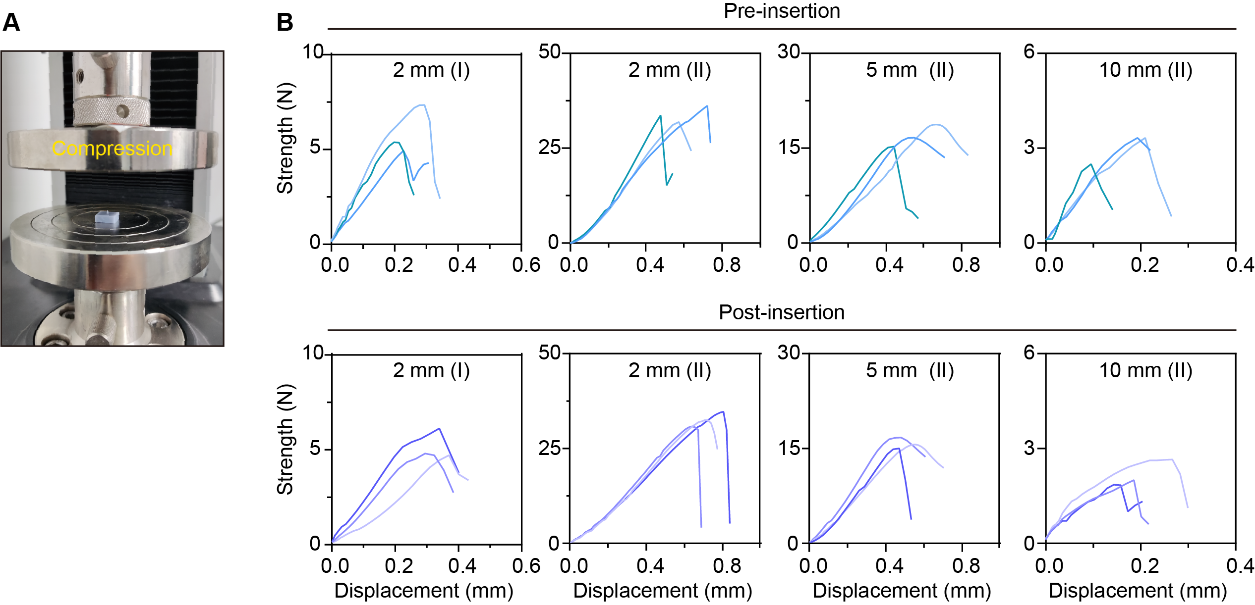
**

**Figure S2.** **Assessment of the mechanical stability *in vivo*.** Compressive strength measurement photograph (A) and curves (B) of the sampling needles with different structural parameters before and after insertion into rats’ dorsal tissue for three days.

**
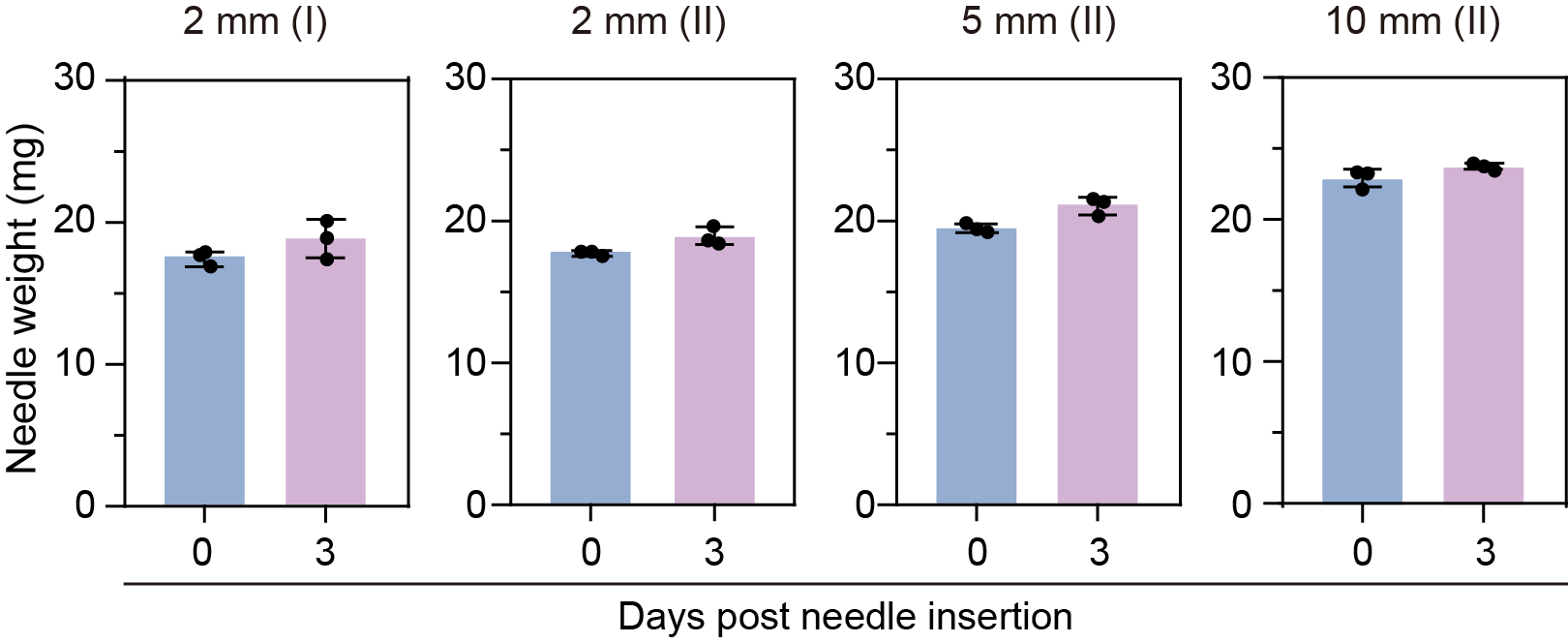
**

**Figure S3.** **Assessment of the needle mass loss.** The needles exhibited no mass loss after the insertion into rats’ dorsal tissues for three days. Data are presented as mean ± SD.

**
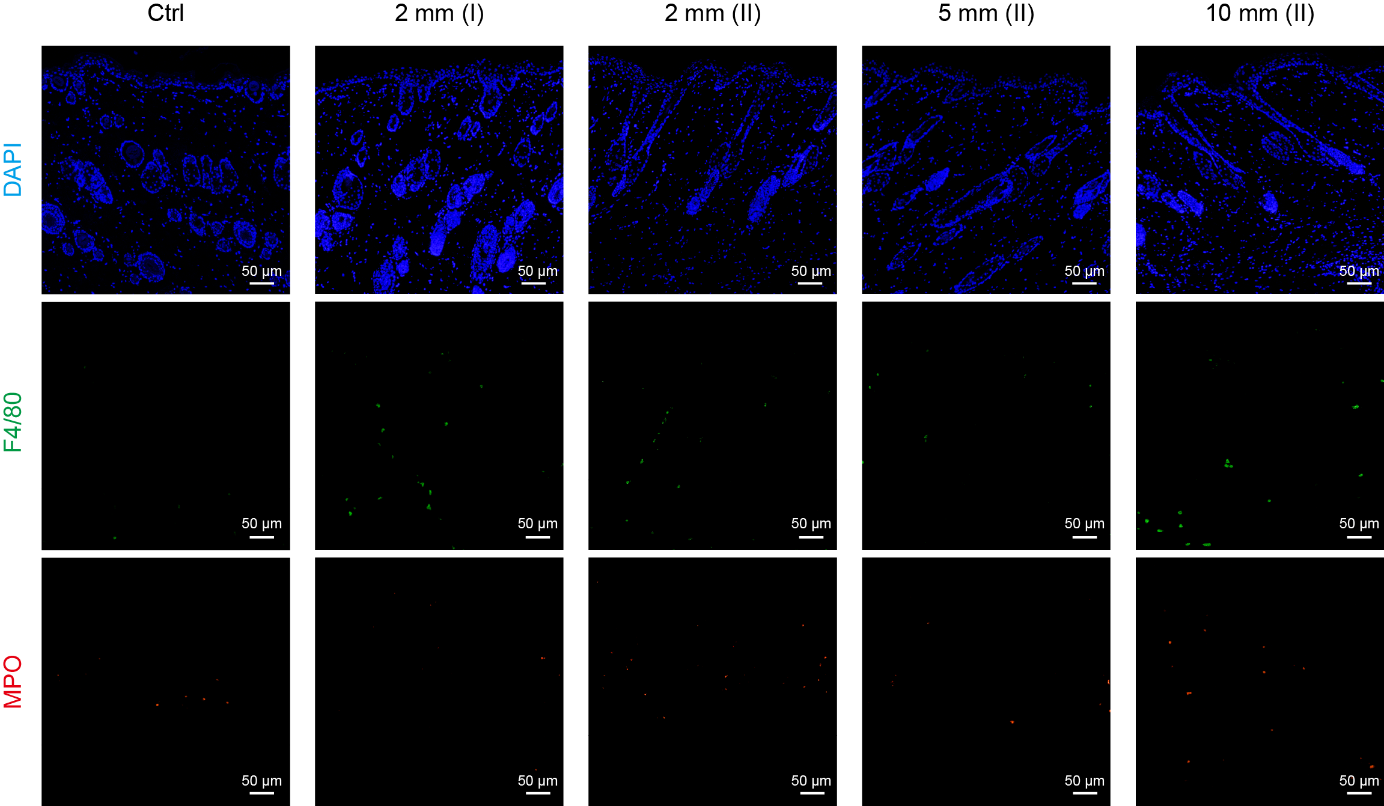
**

**Figure S4.** **Assessment of the inflammatory response.** Representative immunofluorescence staining images of neutrophils (MPO^+^, red) and macrophages (F4/80^+^, green) within the rats' dorsal tissues three days after needle removal.


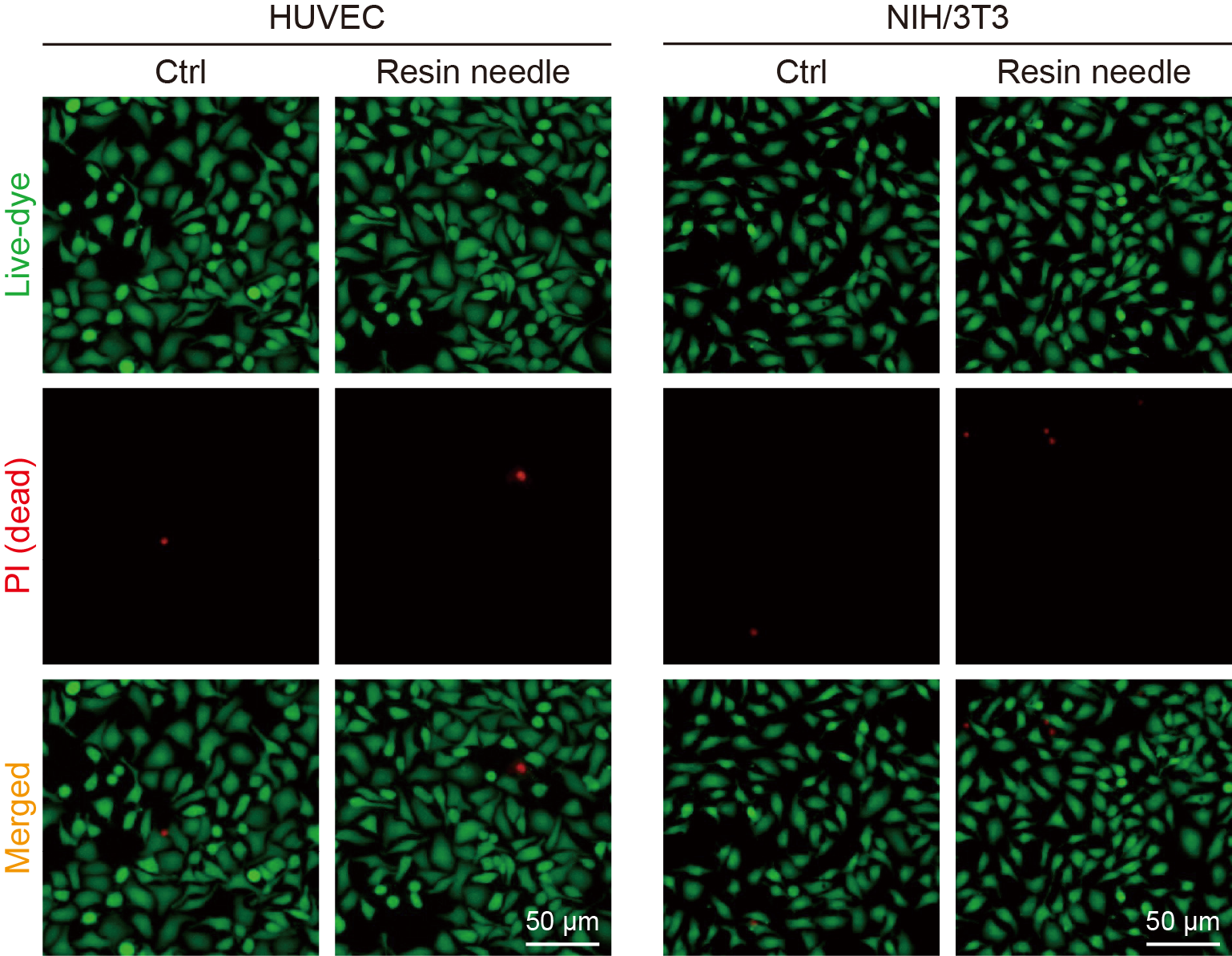


**Figure S5.** **Assessment of device materials’ cytocompatibility.** The Live/Dead staining images show no obvious cell death in both HUVEC and NIH/3T3 cells after incubation with resin needles, suggesting their good cytocompatibility.

**
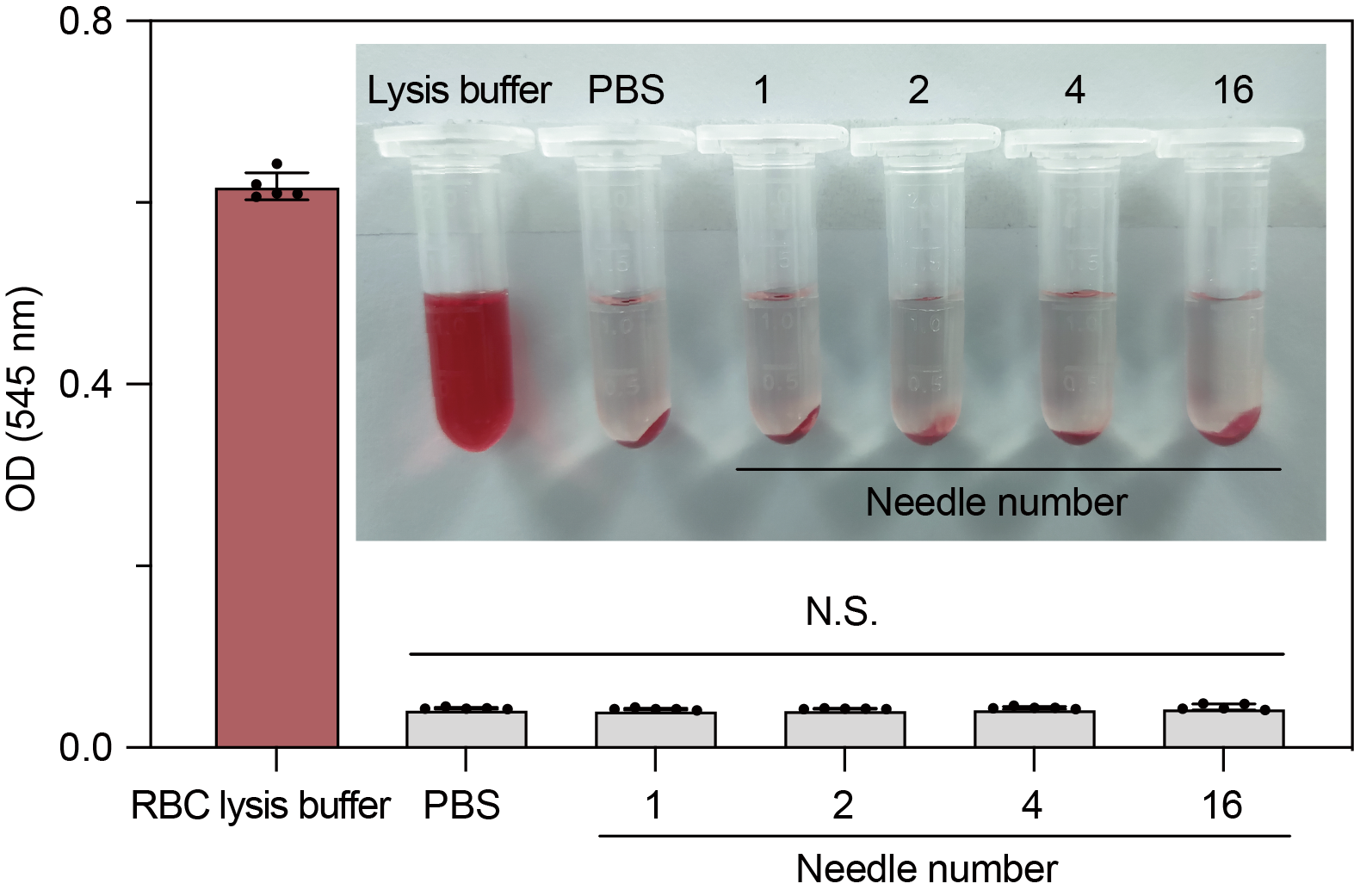
**

**Figure S6.** **The hemolysis activity analysis.** No significant difference was found in the hemolysis assay between the resin sampling needles and PBS treatments, suggesting needles’ good hemocompatibility. Data are presented as mean ± SD. N.S., not significant; one-way ANOVA.


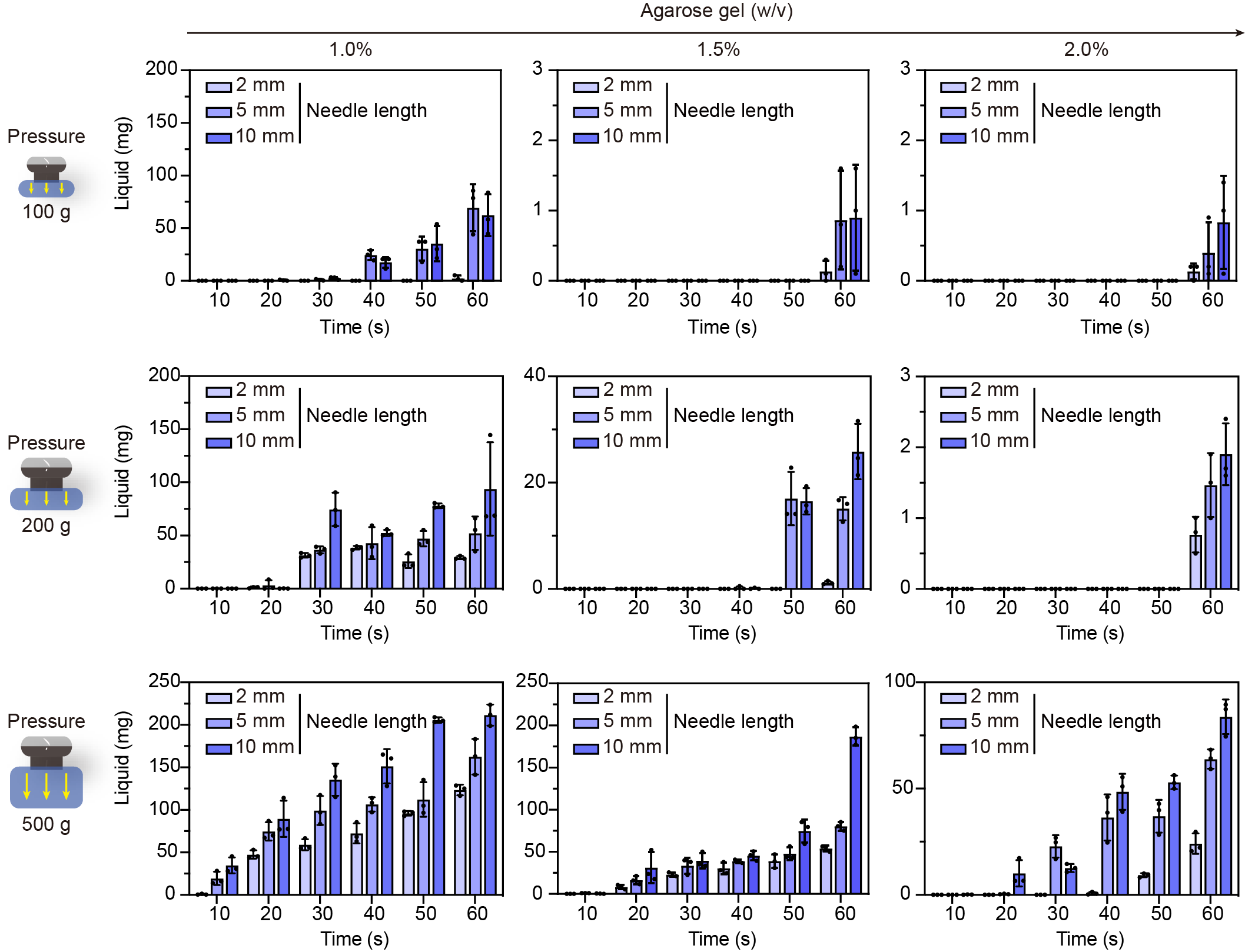


**Figure S7. Sampling efficiency varied by** **pressure, needle length, and agarose mass concentration.** The liquid extraction efficiency is approximately proportional to the force and duration of applied pressure, as well as the needle height, while being inversely proportional to the mass concentration of agarose gel. Data are presented as mean ± SD.

**
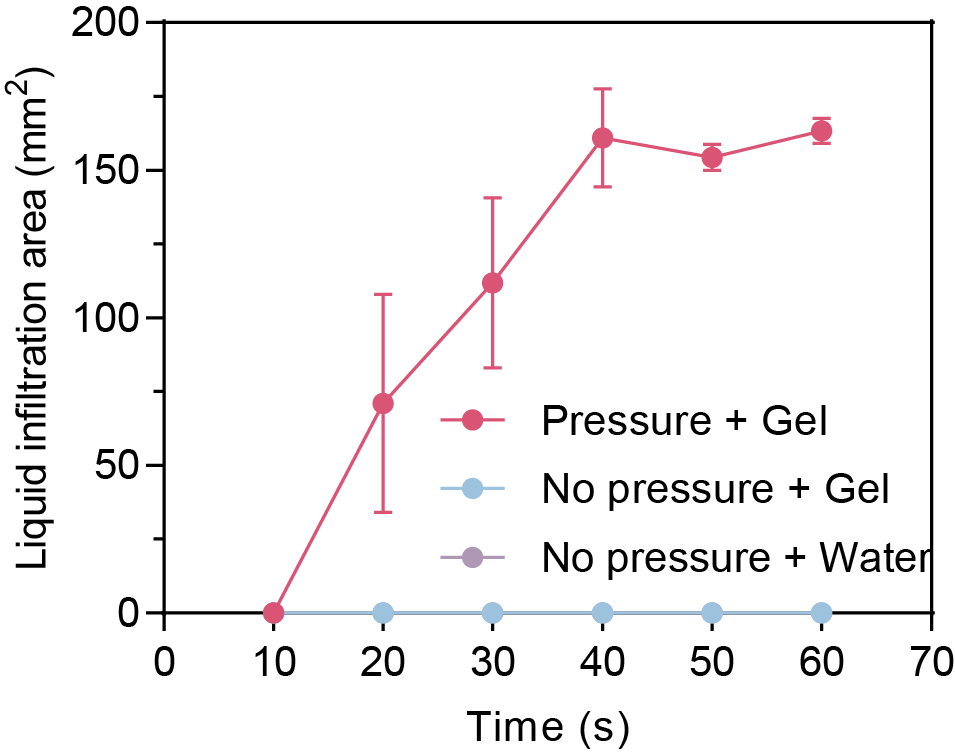
**

**Figure S8. Liquid extraction performance under different conditions.** Little liquid was extracted without pressure applied when the sampling needles were inserted into the gel or pure water, indicating a highly controllable sampling manner that would contribute to preventing undesired contamination on reaction papers. Data are presented as mean ± SD.


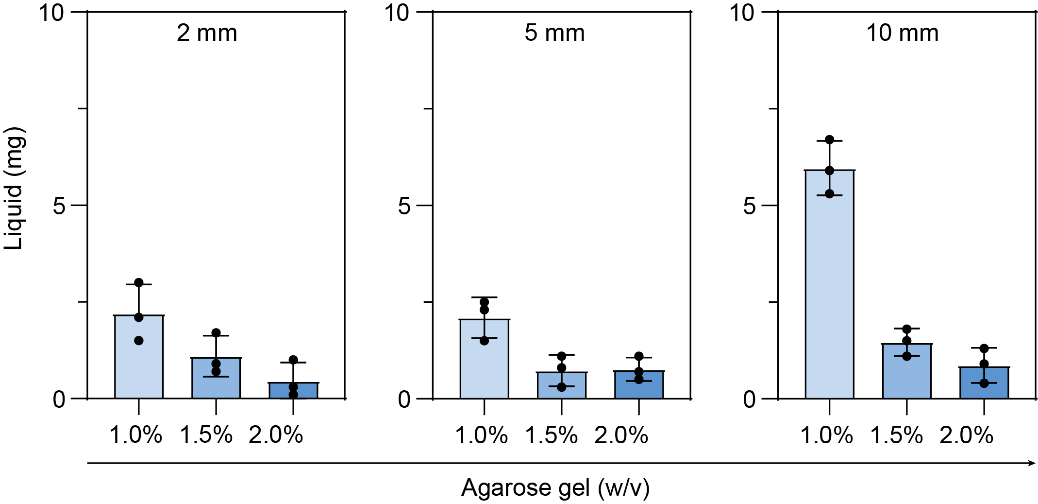


**Figure S9.** **Liquid extraction performance of the device with surface-smoothed needles.** The liquid extraction efficiency of the surface-smoothed needles was much lower than that of the surface-channeled needles across all the agarose gel under the same pressure condition. Data are presented as mean ± SD.

**
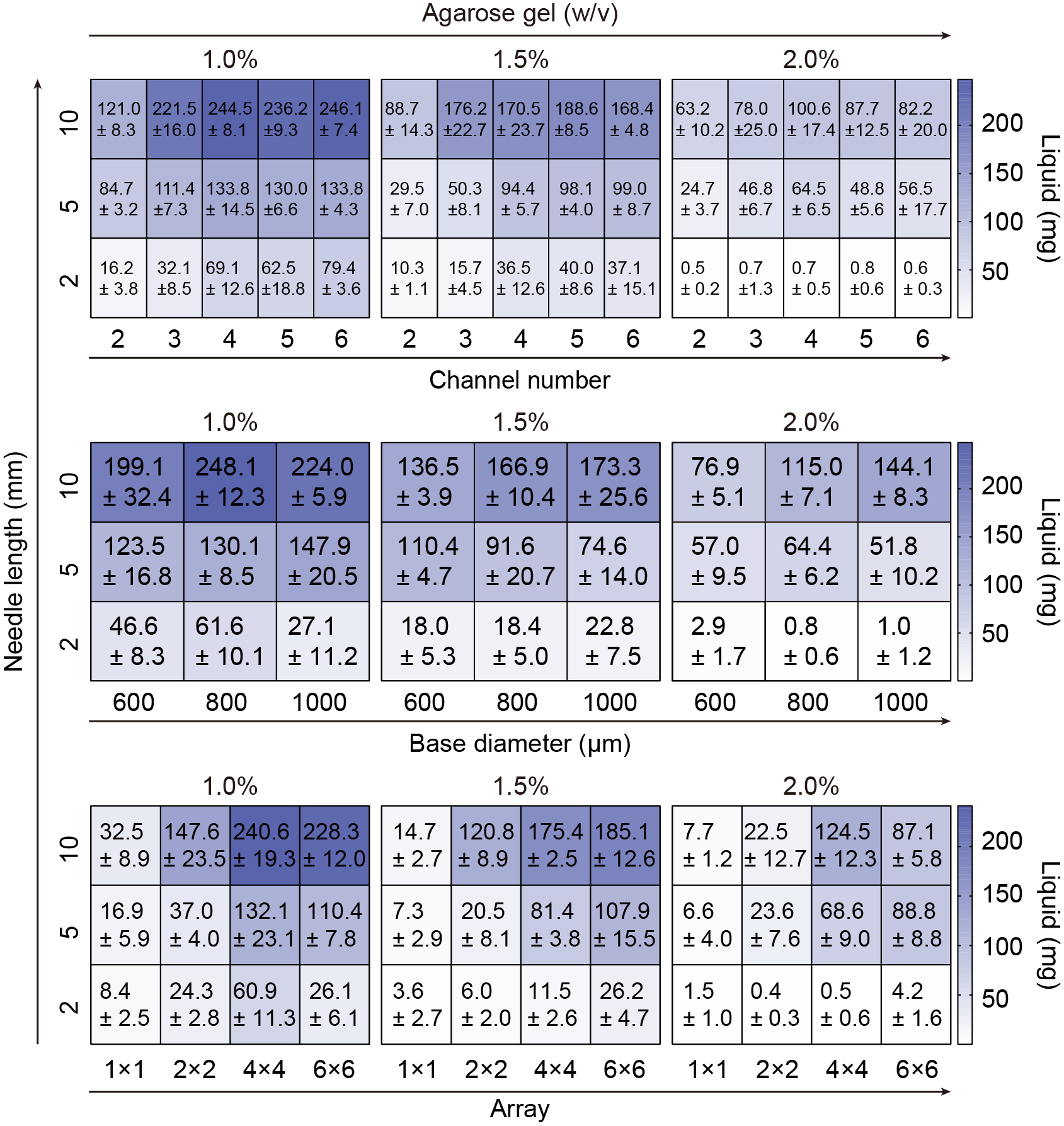
**

**Figure S10. Quantification of liquid extraction using the device with type II sampling needles.** The liquid extraction efficiency of type II sampling needles was also varied by needle and array parameters.

**
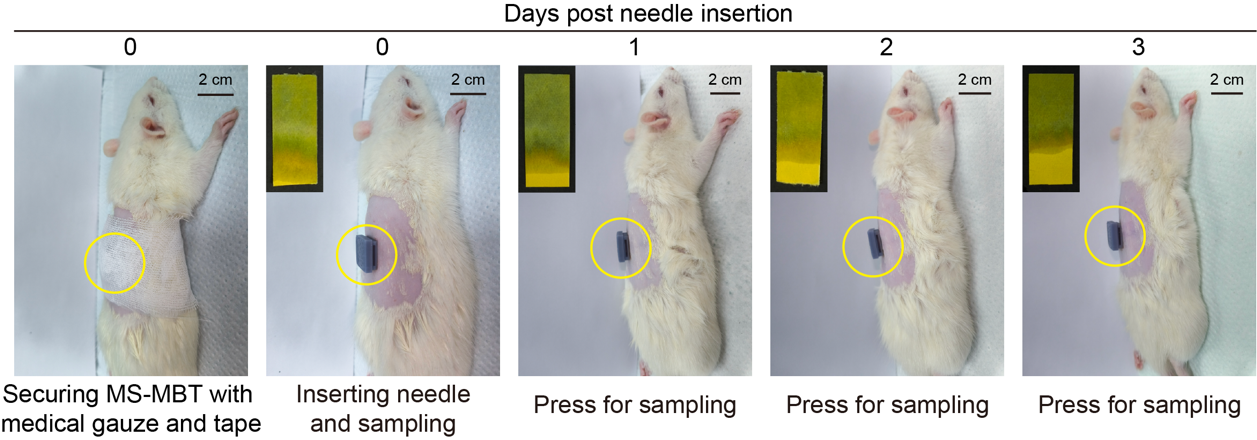
**

**Figure S11. The sustained sampling process with** **the MS-MBT device *in vivo*.** Images show a rat whose dorsal tissue was inserted with sampling needles of the medical gauze/tape-secured MS-MBT device, followed by sampling once daily for three days. The insets show pH test strips obtained from the device after a reaction with the *in vivo* collected liquid samples. Yellow circles highlight the device’s location.

**
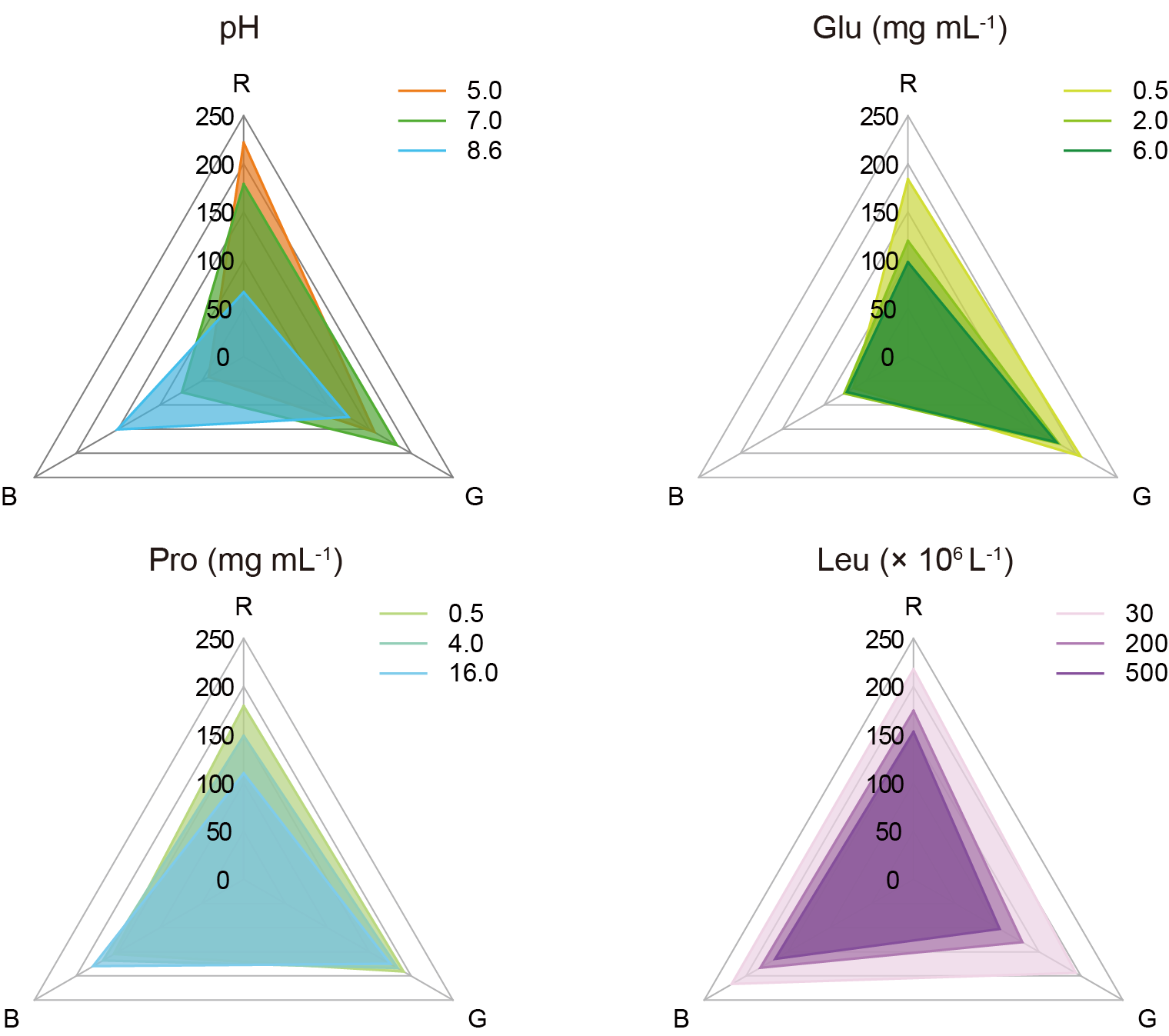
**

**Figure S12.** **Radial graphs of the RGB values of the biochemical reaction papers obtained by ColorPicker readings.** The four indicator reaction papers yielded significantly distinct RGB color values at different values or concentrations.

**
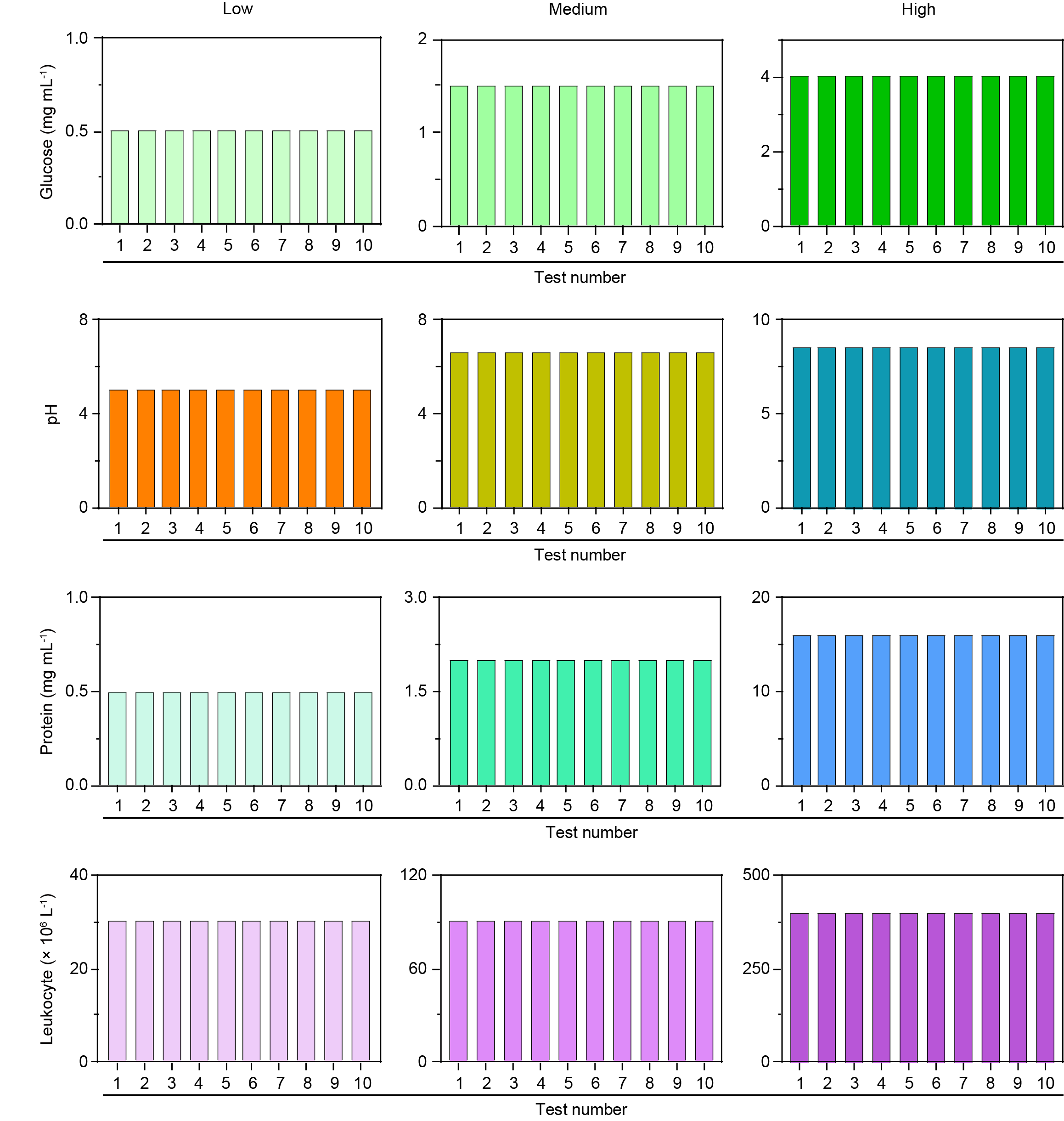
**

**Figure S13. Assessment of the detection stability.** Variation in indicator values or concentrations obtained from ten repeated tests on samples using the MS-MBT device. RSD (relative standard deviation) = 0.

**
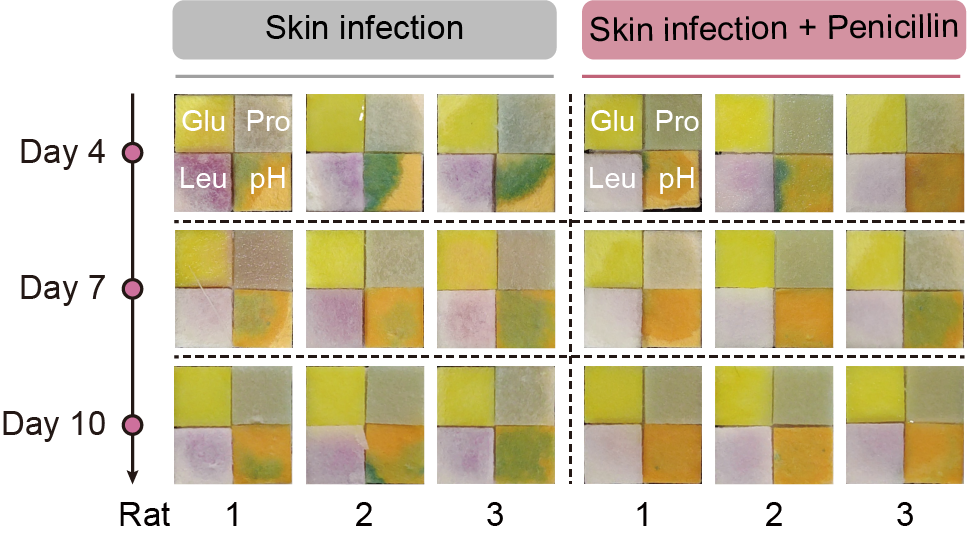
**

**Figure S14. Real-time** **monitoring of** ***S. aureus*-infected deep skin wound in rats.** The color changes of the pH and Leu indicator papers infiltrated with wound exudate exhibited variations among different treatment groups and at the given timepoints.

**
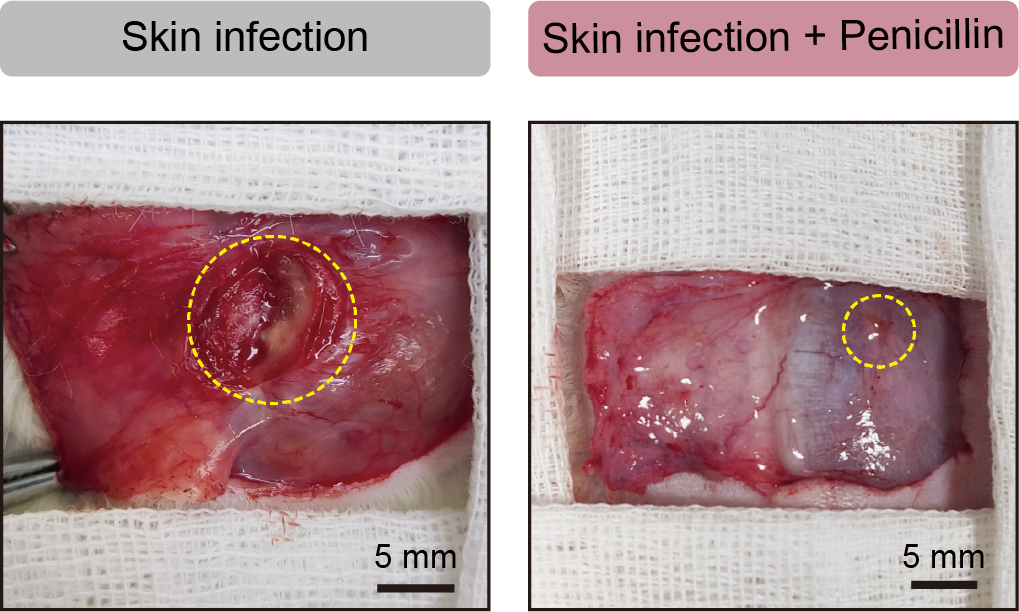
**

**Figure S15.** **The wound images acquired on day ten post-infection.** The untreated rats with skin infection exhibited a more serious inflammatory exudation compared to the penicillin-treated ones. The yellow dotted circles highlight the surgical incision approaching subcutaneous tissue infected with *S. aureus*.

**
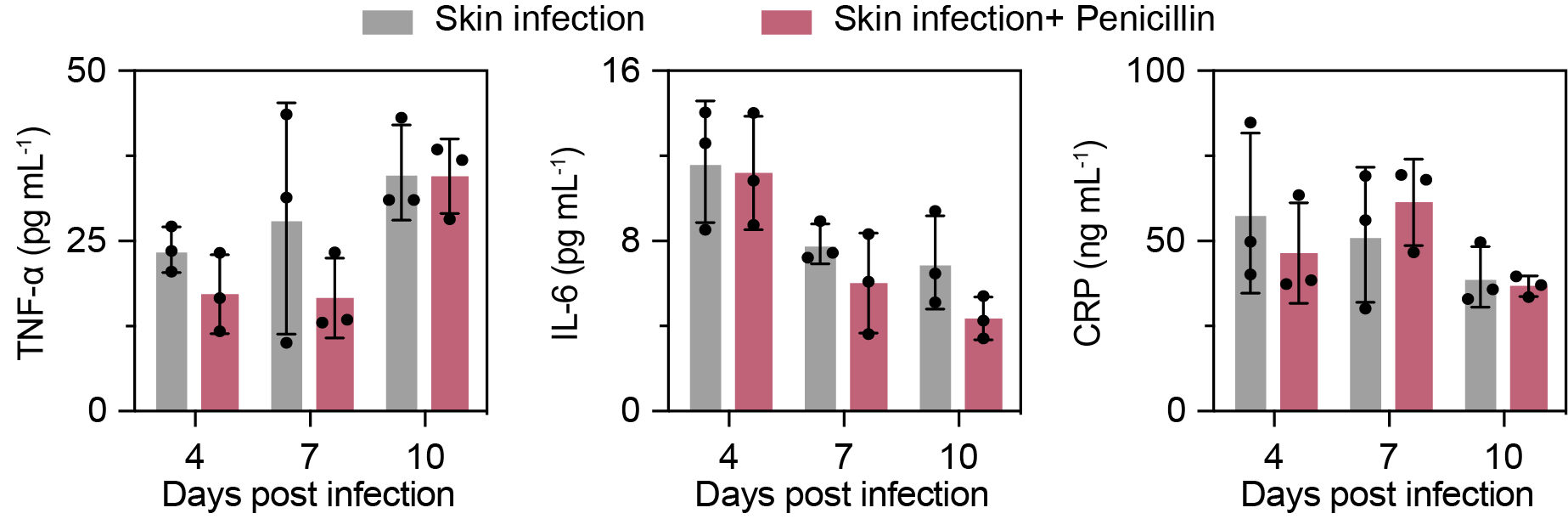
**

**Figure S16. Monitoring of serum inflammatory cytokines from the rats with *S. aureus*-infected skin wound.** The rats revealed no significant alterations in serum TNF-α, IL-6, and CRP levels following either penicillin treatment or across the observation period, suggesting the potential limitations of systemic biomarkers for accurately reflecting the localized wound infection (n = 3). Data are presented as mean ± SD; Student’s *t*-tests.

**
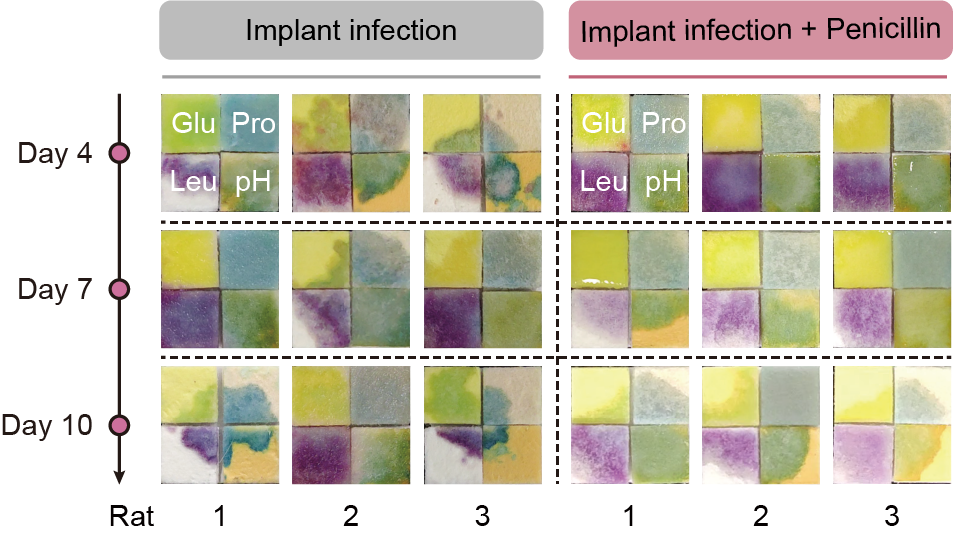
**

**Figure S17.** **Real-time monitoring of hip joint implant infected with *S. aureus* in rats.** The color changes of the pH, Glu, Pro, and Leu indicator papers infiltrated with wound exudate exhibited variations between untreated and penicillin-treated samples and at the given timepoints.

**
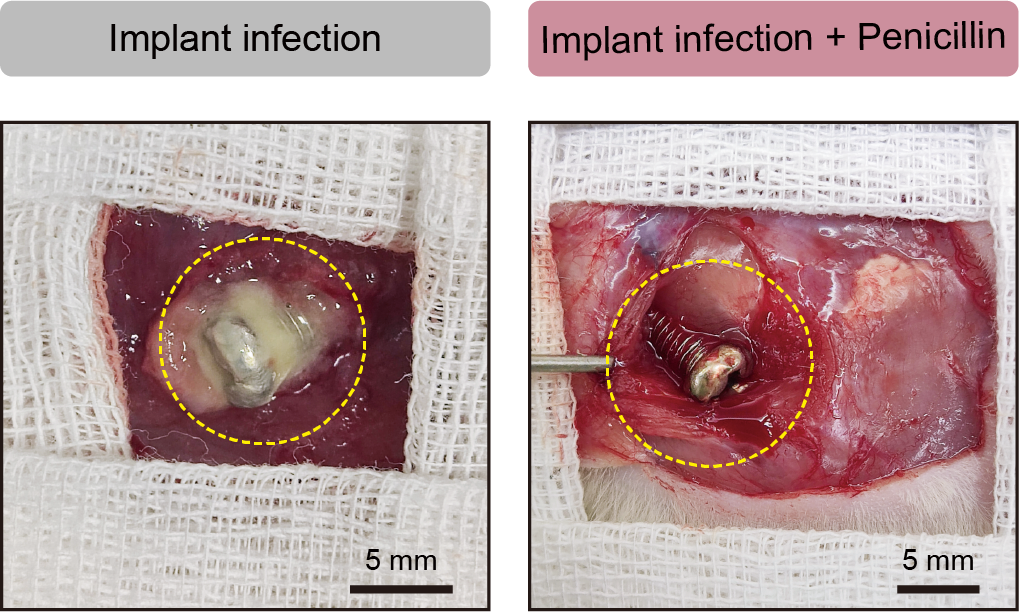
**

**Figure S18. The wound images acquired on day ten post hip joint implant infection.** The untreated rats produced more purulent exudate on the implant than the penicillin-treated rats. The yellow dotted circles highlight the surgical incision to the hip joint where the *S. aureus*-infected implant (stainless-steel screw) was placed.

**
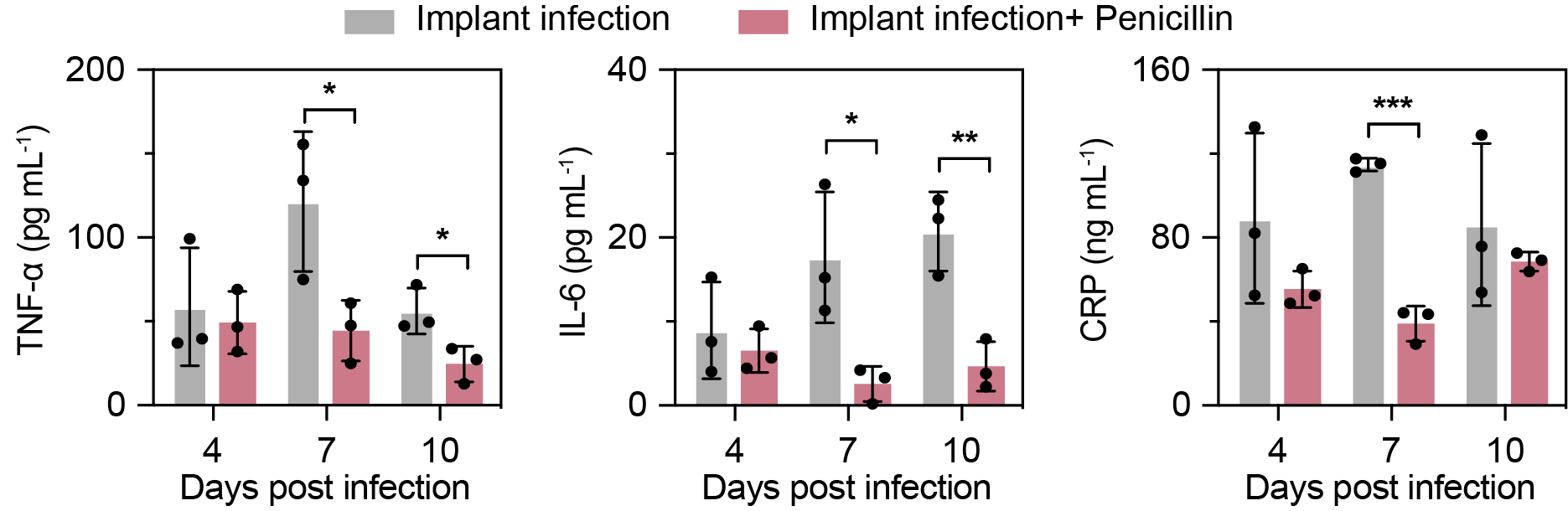
**

**Figure S19. Monitoring of serum inflammatory cytokines from the rats with hip joint implant.** Penicillin treatment effectively reduced the serum TNF-α, IL-6, and CRP levels at the given timepoints (n = 3). Data are presented as mean ± SD. **p < 0.05,* ***p < 0.01, ***p < 0.001*; Student’s *t*-tests.

**
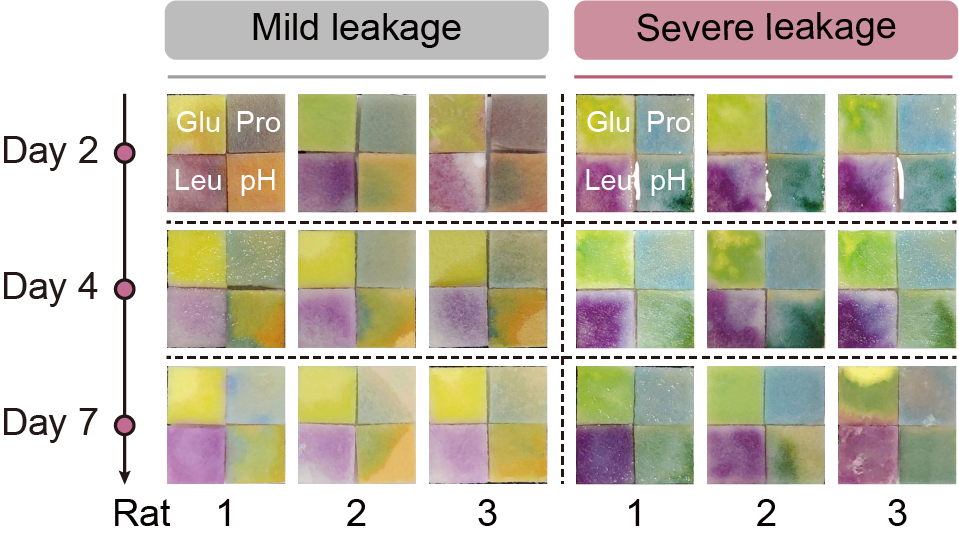
**

**Figure S20. Real-time monitoring of intestinal leakage in rats.** The color changes of the pH, Glu, Pro, and Leu indicator papers infiltrated with intestinal leakage liquid exhibited variations between mild and severe leakages at the given timepoints.

**
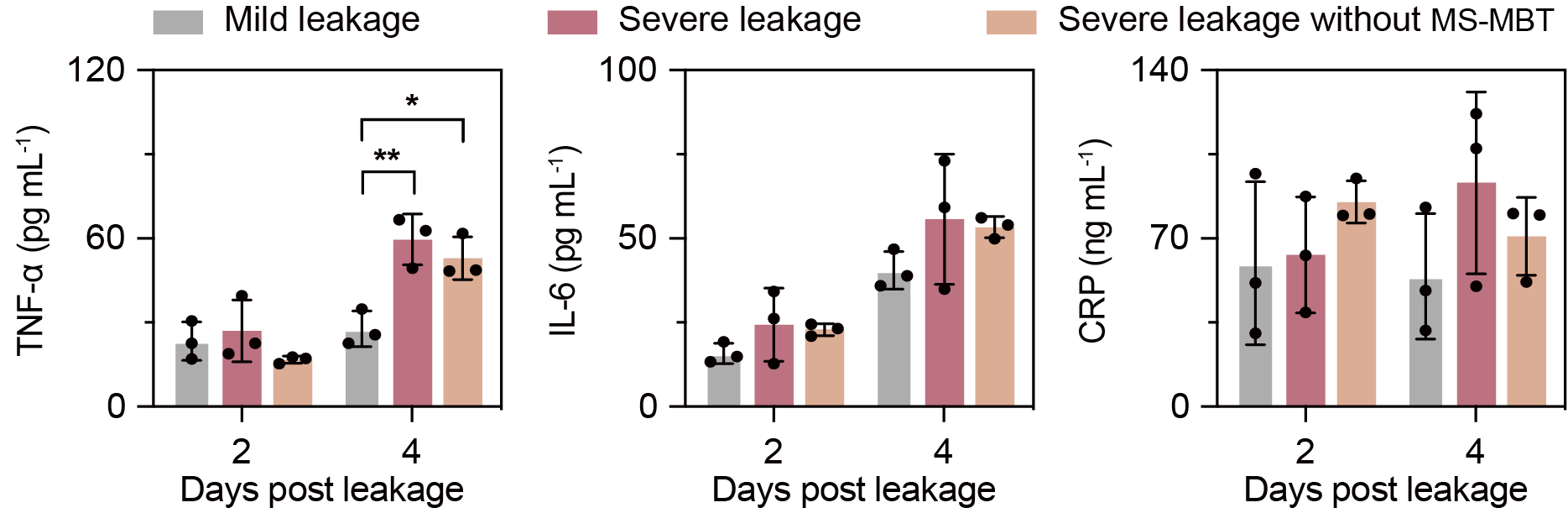
**

**Figure S21. Monitoring of serum inflammatory cytokines from the rats bearing mild or severe intestinal leakage.** The rats bearing severe intestinal leakage either with or without MS-MBT treatment exhibited an increased TNF-α level than the rats bearing mild intestinal leakage with MS-MBT treatment on day four post-surgery (n = 3). Data are presented as mean ± SD. **p < 0.05, **p < 0.01*; one-way ANOVA.

**
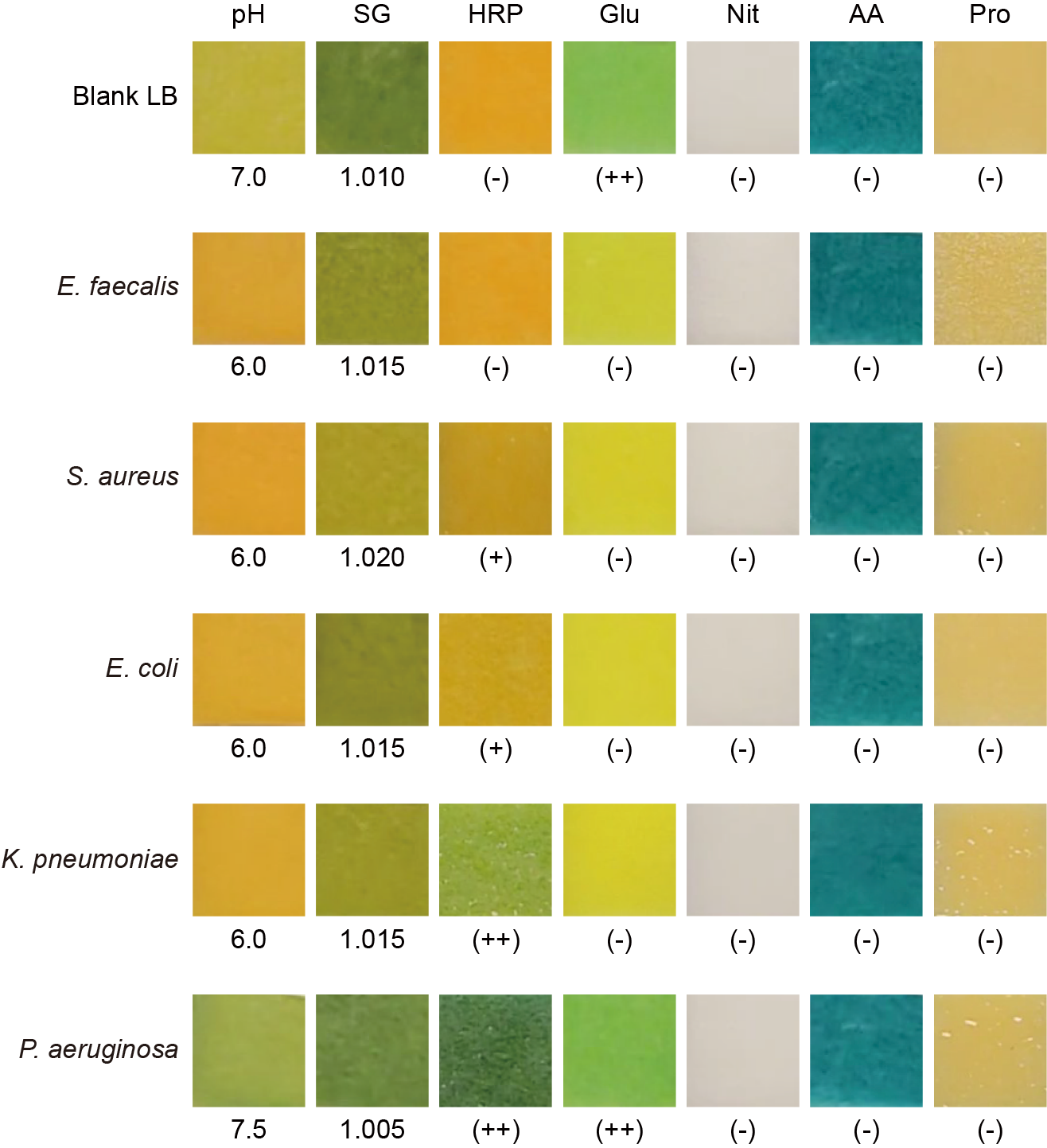
**

**Figure S22. Screening of valuable biochemical indicators for bacterial identification.** pH, HRP, and SG displayed distinct color rendering outcomes across the five bacterial species according to the manual readings using the standard color charts.

**
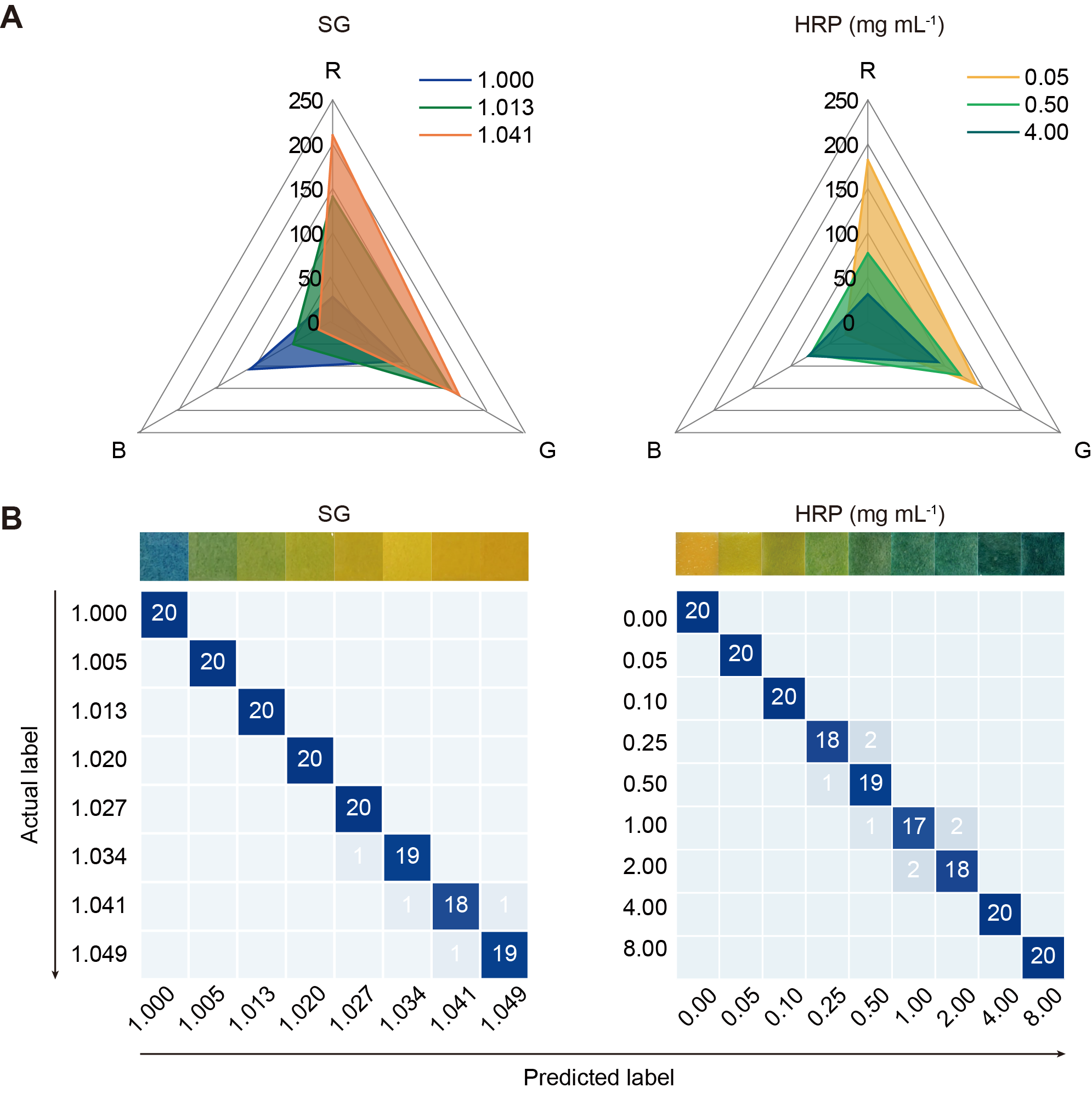
**

**Figure S23. ColorPicker analysis on the SG and HRP indicator papers.** (**A**) Radial graphs showing the RGB values of the reaction papers obtained by ColorPicker readings. (**B**) Confusion matrices visualizing the agreement between the actual and ColorPicker-predicted values for SG and HRP. The two indicator reaction papers yielded significantly distinct RGB color values at different values and could be accurately predicted by ColorPicker.

**
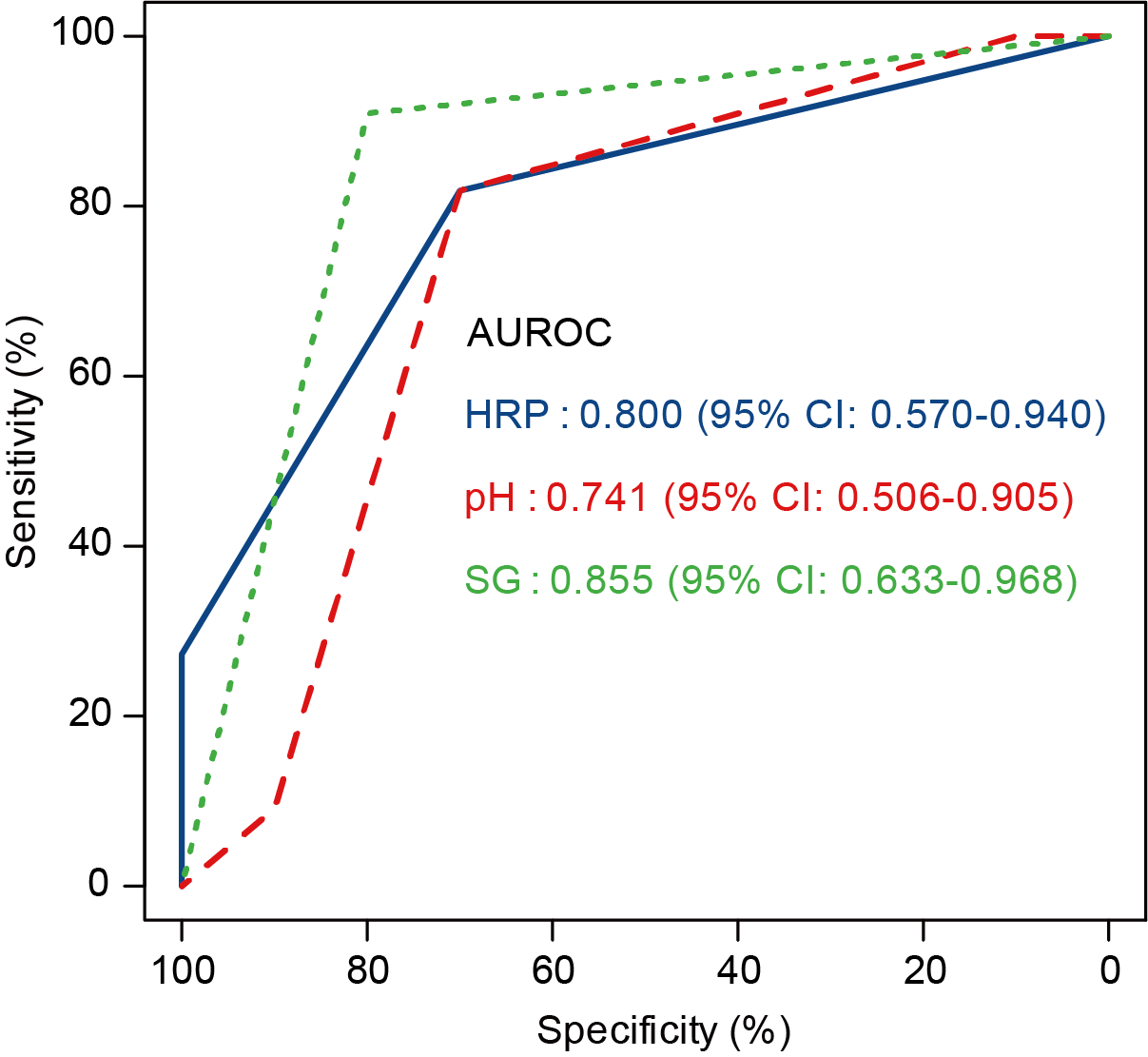
**

**Figure S24.** **The ROC curves and AUROC values on** **differentiating *E. coli*- and *S. aureus* infection in rats with the individual indicators.** Single-use of HRP, pH, or SG was less effective in differentiating the two bacteria than the combination of the three indicators.

**Table S1.** The quantification of liquid extraction using the device with different sample needles in the waterproof membrane-covered agarose gels.

| **Agarose gel (w/v)** | **Channel number** | **2 mm (I)** | **5 mm (I)** | **10 mm (I)** | **2 mm (II)** | **5 mm (II)** | **10 mm (II)** |
| --- | --- | --- | --- | --- | --- | --- | --- |
| **1.0%** | **1** | 20.6 ± 3.0 | 20.3 ± 2.3 | 32.7 ± 6.4 | 15.1 ± 4.3 | 43.4 ± 5.3 | 88.5 ± 7.4 |
|  | **2** | 32.0 ± 7.4 | 39.4 ± 9.7 | 55.7 ± 13.7 | 16.2 ± 3.8 | 84.7 ± 3.2 | 121.0 ± 8.3 |
|  | **3** | 66.0 ± 9.1 | 108.4 ± 9.0 | 170.3 ± 18.4 | 32.1 ± 8.5 | 111.4 ± 7.3 | 221.5 ± 16.0 |
|  | **4** | 158.8 ± 11.5 | 121.8 ± 11.6 | 185.4 ± 14.3 | 69.1 ± 12.6 | 133.8 ± 14.5 | 244.5 ± 8.1 |
|  | **5** | 137.3 ± 14.6 | 142.6 ± 29.5 | 191.8 ± 12.8 | 62.5 ± 18.8 | 129.9 ± 6.6 | 236.2 ± 9.3 |
|  | **6** | 125.9 ± 18.2 | 130.6 ± 13.2 | 199.2 ± 14.6 | 79.4 ± 3.6 | 133.8 ± 4.3 | 246.1 ± 7.4 |
| **1.5%** | **1** | 3.5 ± 3.4 | 2.1 ± 1.7 | 39.8 ± 8.2 | 5.7 ± 2.5 | 30.9 ± 10.7 | 72.4 ± 8.6 |
|  | **2** | 3.8 ± 1.5 | 21.6 ± 2.8 | 63.2 ± 11.4 | 10.3 ± 1.1 | 29.5 ± 7.0 | 88.7 ± 14.3 |
|  | **3** | 22.3 ± 5.6 | 59.2 ± 10.3 | 97.7 ± 21.5 | 15.7 ± 4.5 | 50.3 ± 8.1 | 176.2 ± 22.7 |
|  | **4** | 22.8 ± 6.5 | 53.9 ± 10.2 | 102.4 ± 4.3 | 36.5 ± 12.6 | 94.4 ± 5.7 | 170.5 ± 23.7 |
|  | **5** | 25.4 ± 4.4 | 45.0 ± 4.6 | 97.0 ± 13.7 | 40.0 ± 8.6 | 98.1 ± 3.9 | 188.6 ± 8.5 |
|  | **6** | 16.8 ± 3.8 | 53.0 ± 7.0 | 107.1 ± 16.9 | 37.1 ± 15.1 | 99.0 ± 8.7 | 168.4 ± 4.8 |
| **2.0%** | **1** | 1.1 ± 0.8 | 0.3 ± 0.2 | 20.3 ± 3.0 | 0.1 ± 0.2 | 20.0 ± 2.3 | 45.0 ± 8.1 |
|  | **2** | 3.1 ± 2.0 | 0.5 ± 0.3 | 27.0 ± 10.3 | 0.5 ± 0.2 | 24.7 ± 3.7 | 63.3 ± 10.2 |
|  | **3** | 4.4 ± 2.0 | 25.5 ± 6.5 | 38.9 ± 8.7 | 0.7 ± 1.3 | 46.8 ± 6.7 | 78.0 ± 25.0 |
|  | **4** | 17.1 ± 6.9 | 26.5 ± 2.2 | 49.0 ± 5.5 | 0.7 ± 0.5 | 64.5 ± 6.5 | 100.6 ± 17.4 |
|  | **5** | 7.0 ± 1.8 | 32.2 ± 5.5 | 52.9 ± 5.5 | 0.8 ± 0.6 | 48.8 ± 5.6 | 87.7 ± 12.5 |
|  | **6** | 9.3 ± 3.0 | 24.2 ± 5.2 | 60.1 ± 4.1 | 0.6 ± 0.3 | 56.5 ± 17.7 | 82.2 ± 20.0 |
| **Agarose gel (w/v)** | **Base diameter (μm)** | **2 mm (I)** | **5 mm (I)** | **10 mm (I)** | **2 mm (II)** | **5 mm (II)** | **10 mm (II)** |
| **1.0%** | **600** | 49.8 ± 8.7 | 136.5 ± 10.7 | 196.9 ± 5.5 | 46.6 ± 8.3 | 123.5 ± 16.8 | 199.1 ± 32.4 |
|  | **800** | 134.1 ± 8.8 | 160.7 ± 13.5 | 200.7 ± 7.4 | 61.6 ± 10.1 | 130.1 ± 8.5 | 248.1 ± 12.3 |
|  | **1000** | 103.9 ± 7.2 | 137.1 ± 9.4 | 184.0 ± 6.4 | 27.1 ± 11.2 | 147.9 ± 20.5 | 224.0 ± 5.9 |
| **1.5%** | **600** | 42.1 ± 5.6 | 53.7 ± 4.8 | 127.3 ± 10.6 | 18.0 ± 5.3 | 110.4 ± 4.7 | 136.5 ± 3.9 |
|  | **800** | 40.3 ± 3.4 | 75.1 ± 3.8 | 144.1 ± 18.6 | 18.4 ± 5.0 | 91.6 ± 20.6 | 166.9 ± 10.4 |
|  | **1000** | 36.9 ± 9.5 | 66.5 ± 11.0 | 140.6 ± 9.2 | 22.8 ± 7.5 | 74.6 ± 14.0 | 173.3 ± 25.6 |
| **2.0%** | **600** | 1.2 ± 0.2 | 21.8 ± 6.4 | 55.9 ± 7.1 | 2.9 ± 1.7 | 57.0 ± 9.5 | 76.9 ± 5.1 |
|  | **800** | 20.8 ± 4.6 | 43.6 ± 8.7 | 79.5 ± 2.8 | 0.8 ± 0.6 | 64.4 ± 6.2 | 115.0 ± 7.1 |
|  | **1000** | 15.0 ± 4.8 | 39.3 ± 7.8 | 76.7 ± 13.9 | 1.0 ± 1.2 | 51.8 ± 10.2 | 144.1 ± 8.3 |
| **Agarose gel (w/v)** | **Array** | **2 mm (I)** | **5 mm (I)** | **10 mm (I)** | **2 mm (II)** | **5 mm (II)** | **10 mm (II)** |
| **1.0%** | **1 × 1** | 3.6 ± 1.8 | 16.1 ± 3.1 | 38.9 ± 7.7 | 8.4 ± 2.5 | 16.9 ± 5.9 | 32.5 ± 8.9 |
|  | **2 × 2** | 51.8 ± 11.1 | 98.3 ± 18.6 | 138.6 ± 5.3 | 24.3 ± 2.8 | 37.0 ± 4.0 | 147.6 ± 23.5 |
|  | **4 × 4** | 112.9 ± 21.4 | 169.4 ± 13.6 | 212.3 ± 7.8 | 60.9 ± 11.3 | 132.1 ± 23.1 | 240.6 ± 19.3 |
|  | **6 × 6** | 125.9 ± 3.2 | 212.7 ± 13.2 | 222.8 ± 22.9 | 26.1 ± 6.1 | 110.4 ± 7.8 | 228.3 ± 12.0 |
| **1.5%** | **1 × 1** | 1.7 ± 0.6 | 9.5 ± 2.3 | 20.0 ± 2.6 | 3.6 ± 2.7 | 7.3 ± 2.9 | 14.7 ± 2.7 |
|  | **2 × 2** | 3.8 ± 2.6 | 30.3 ± 8.2 | 53.9 ± 13.1 | 6.0 ± 2.0 | 20.5 ± 8.1 | 120.8 ± 9.0 |
|  | **4 × 4** | 46.3 ± 5.9 | 73.4 ± 6.8 | 156.5 ± 42.7 | 11.5 ± 2.6 | 81.4 ± 3.8 | 175.4 ± 2.5 |
|  | **6 × 6** | 21.5 ± 1.9 | 144.5 ± 8.1 | 178.9 ± 22.0 | 26.2 ± 4.7 | 107.9 ± 15.5 | 185.1 ± 12.6 |
| **2.0%** | **1 × 1** | 0.5 ± 0.1 | 3.2 ± 0.7 | 4.1 ± 1.3 | 1.5 ± 1.0 | 6.6 ± 4.0 | 7.7 ± 1.2 |
|  | **2 × 2** | 0.7 ± 0.5 | 8.6 ± 2.9 | 15.2 ± 3.6 | 0.4 ± 0.3 | 23.6 ± 7.6 | 22.5 ± 12.7 |
|  | **4 × 4** | 22.7 ± 7.6 | 56.6 ± 13.5 | 78.3 ± 11.2 | 0.5 ± 0.6 | 68.6 ± 9.0 | 124.5 ± 12.3 |
|  | **6 × 6** | 12.7 ± 2.4 | 70.4 ± 15.1 | 104.4 ± 13.5 | 4.2 ± 1.6 | 88.8 ± 8.8 | 87.1 ± 5.8 |

**Table S2.** The reaction principles and corresponding color-changing patterns.

| Indicator | Abbreviation | Reaction principle | Color changing pattern |
| --- | --- | --- | --- |
| pH | pH | Acid-base indicators | Orange→green→blue |
| Glucose | Glu | Glucose oxidase-peroxidase | Yellow→green |
| Protein | Pro | Protein error of pH indicators | Yellow→blue-green |
| Leukocyte | Leu | Esterase | White→purple |
| Specific gravity | SG | Polyelectrolyte ion depolymerization | Blue→green→yellow |
| Horse radish peroxidase | HRP | Tetramethylbenzidine oxidation | Yellow→green |

**Table S3.** Comparison of measured values between the MS-MBT device and clinically standardized quantitative methods.

| **Indicator** | **Sample** | **MS-MBT** | **Clinical methods** | **Relative error (%)** |
| --- | --- | --- | --- | --- |
| **Glucose (mg/mL)** | 1 | 0.5 | 0.52^a)^ | 3.85 |
|  | 2 | 1.5 | 1.57^a)^ | 4.46 |
|  | 3 | 2 | 2.03^a)^ | 1.48 |
| **Protein (mg/mL)** | 1 | 1 | < LOD^a)^ | / |
|  | 2 | 4 | 4.02^a)^ | 0.50 |
|  | 3 | 16 | 16.25^a)^ | 1.54 |
| **Leukocyte (× 10^6^ L^-1^)** | 1 | 60 | 65^b)^ | 7.69 |
|  | 2 | 300 | 310^b)^ | 3.23 |
|  | 3 | 500 | 490^b)^ | 2.04 |

^a)^ Data obtained from an automated hematology analyzer (Mindray BC-5390 CRP).

^b)^ Data obtained from an automated biochemical analyzer (Abbott ARCHITECT C16000 System).

**Table S4.** Comparisons of wound monitoring approaches based on our MS-MBT device and other microneedle array devices.

| **Name/Form** | **Material** | **Fabrication** | **Detection depth** | **Detection indicator** | **Sensor type** | **Data reading** | **Application scenario** | **Advantages** | **Ref** |
| --- | --- | --- | --- | --- | --- | --- | --- | --- | --- |
| FM@ST microneedle patch | SF^a)^, TA^b)^ | Reverse molding | 600 μm | H_2_O_2_ (39 nM~100 μM) | Fluorescence probe | Fluorescence imaging | *S. aureus*-induced myositis in mice | Integrated antibacterial treatment and monitoring | ^[1]^ |
| MXene-based microneedle patch | Mxene, SF^a)^, PU^c)^, SP^d)^ | One-step reverse molding | 500~1000 μm | Motion | Electrical impedance sensor | RMS multimeter | Circular cutaneous wounds in mice | Easy operation, biomimetic design, high drug loading efficiency, and NIR-trigger drug rapid release | ^[2]^ |
| Double-layer microneedle patch | SF^a)^, MA^e)^, PVA^f)^ | Layered casting | 800 μm | pH (4~9) | pH-sensitive fluorescent indicators | Machine learning-assisted image analysis | Bacterial infection wounds in rats | Integrated antibacterial treatment and monitoring, high predictive accuracy | ^[3]^ |
| Encoded structural color microneedle patch | PEGDA^g)^ | Partitioned and layered casting | - | pH (4~8),  glucose (1~4 mg/mL),  histamine (1~8 μg/mL) | Photonic crystals | Reflection spectrum analyzer | Bacterial infection and diabetic wounds in rats | Multiple detection, little exudate required, wide medical application | ^[4]^ |
| Biomimetic porous microneedle patch | PCLMA^h)^, Hep-SH-HAMA^i)^ | Digital light processing 3D printing | 1000 μm | pH | Phenol red colorimetry | Naked eyes | Infection-induced chronic wounds in rats | Integrated rapid sampling, diagnosis, and dual, on-demand therapy capabilities | ^[5]^ |
| Superstructure microfluidic microneedle chip | SF^a)^, PC^j)^, MXene | One-step reverse molding | 0.3~2 mm | pH (4~9),  motion,  inflammatory factors | H^+^/OH^-^-responsive PC film,  Electrical impedance sensor,  fluorescent antibody | Reflection spectrum analyzer,  RMS multimeter,  fluorescence imaging | Circular cutaneous wounds in mice | Multifunctional capabilities, adaptability, enhanced fluid control | ^[6]^ |
| 3D origami microneedle patch | PU^c)^, SF^a)^, MXene | Casting and reverse molding | 1000 μm | pH (6~8),  glucose (0~10 mM),  motion | pH sensing reagents,  glucose oxidase,  electrical impedance sensor | The grayscale value and color model,  RMS multimeter | Bar cut wounds in mice | Easy, rapid, and abundant production; integrating biochemical sensing detection, controllable drug release, and motion sensing | ^[7]^ |
| MS-MBT device | Resin | UV-crosslinked 3D printing | 2-10 mm | pH (4.6~8.6),  glucose (0~6 mg/mL),  protein (0~16 mg/mL),  leukocyte (15~500 **×** 10^6^ L^-1^) | Dry-chemical reaction papers | Smartphone-accessible image processing software | *S. aureus*-infected deep skin injury and surgical implantation wounds, postoperative intestinal leakages in rats | Easy fabrication process, integrated deep sampling and continuous multiplex biochemical testing, convenient result reading, multiple wound monitoring | This study |

^a)^ Silk fibroin, ^b)^ Tannic acid, ^c)^ Polyurethane, ^d)^ Spidroin, ^e)^ Methylacrylate, ^f)^ Polyvinyl alcohol, ^g)^ Polyethylene glycol diacryla mide, ^h)^ Methacrylated polycaprolactone, ^i)^ Thiolated heparin-methacryloylated hyaluronic acid, ^j)^ Photonic crystal.

References

[1] J. Ouyang, L. Sun, Z. She, R. Li, F. Zeng, Z. Yao, S. Wu, *ACS Appl. Mater. Interfaces* **2024**.

[2] H. Lu, W. Shao, B. Gao, S. Zheng, B. He, *Acta Biomater.* **2023**, *159*, 201.

[3] J. Xiao, Z. Zhou, G. Zhong, T. Xu, X. Zhang, *Adv. Funct. Mater.* **2024**, *34* (22).

[4] M. Lu, X. Zhang, D. Xu, N. Li, Y. Zhao, *Adv. Mater.* **2023**, *35* (19), e2211330.

[5] Y. Liu, C. He, T. Qiao, G. Liu, X. Li, Q. Wan, Z. Zhu, Y. He, *Adv. Funct. Mater.* **2024**, *34* (24).

[6] Q. Zhou, K. Dong, M. Wei, B. He, B. Gao, *Adv. Funct. Mater.* **2024**, *34* (25).

[7] Y. Wang, B. Gao, B. He, *Small* **2023**, *19* (3).

**Supporting video captions**

Video S1 Continuously drawing the reaction strip.

Video S2 The liquid extraction performance of the device with surface-channeled needles under different scenarios.

Video S3 The liquid extraction performance of the device with channeled needles or non-channeled needles.

Video S4 Continuous sampling and biochemical testing from the mixed gel and porcine skin tissue, respectively.

Video S5 Local exudate sampling and testing of *S. aureus*-infected deep wound *in vivo*.

Video S6 Local intestinal leakage liquid sampling and testing*.*
